# Coherent spin qubit shuttling through germanium quantum dots

**DOI:** 10.1038/s41467-024-49358-y

**Published:** 2024-07-08

**Authors:** Floor van Riggelen-Doelman, Chien-An Wang, Sander L. de Snoo, William I. L. Lawrie, Nico W. Hendrickx, Maximilian Rimbach-Russ, Amir Sammak, Giordano Scappucci, Corentin Déprez, Menno Veldhorst

**Affiliations:** 1 QuTech and Kavli Institute of Nanoscience, Delft University of Technology, PO Box 5046, 2600 GA Delft, The Netherlands; 2 QuTech and Netherlands Organisation for Applied Scientific Research (TNO), 2628 CK Delft, The Netherlands

**Keywords:** Quantum information, Quantum dots, Qubits

## Abstract

Quantum links can interconnect qubit registers and are therefore essential in networked quantum computing. Semiconductor quantum dot qubits have seen significant progress in the high-fidelity operation of small qubit registers but establishing a compelling quantum link remains a challenge. Here, we show that a spin qubit can be shuttled through multiple quantum dots while preserving its quantum information. Remarkably, we achieve these results using hole spin qubits in germanium, despite the presence of strong spin-orbit interaction. In a minimal quantum dot chain, we accomplish the shuttling of spin basis states over effective lengths beyond 300 microns and demonstrate the coherent shuttling of superposition states over effective lengths corresponding to 9 microns, which we can extend to 49 microns by incorporating dynamical decoupling. These findings indicate qubit shuttling as an effective approach to route qubits within registers and to establish quantum links between registers.

## Abstract (web summary)

Spin shuttling is a promising technique for establishing a quantum link between qubit registers and has been studied in several quantum dot qubit platforms. Here the authors realize coherent shuttling of a hole spin qubit in a minimal quantum dot chain in germanium despite strong spin-orbit coupling.

## Introduction

The envisioned approach for semiconductor spin qubits towards fault-tolerant quantum computation centers on the concept of quantum networks, where qubit registers are interconnected via quantum links^1^. Significant progress has been made in controlling few-qubit registers^2,3^. Recent efforts have led to demonstrations of high fidelity single- and two-qubit gates^4–7^, quantum logic above one Kelvin^8–10^ and operation of a 16 quantum dot array^11^. However, scaling up to larger qubit numbers requires changes in the device architecture^12,13^.

Inclusion of short-range and mid-range quantum links could be particularly effective to establish scalability, addressability, and qubit connectivity. The coherent shuttling of electron or hole spins is an appealing concept for the integration of such quantum links in spin qubit devices. Short-range coupling, implemented by shuttling a spin qubit through quantum dots in an array, can provide flexible qubit routing and local addressability^14,15^. Moreover, it allows to increase connectivity beyond nearest-neighbor coupling and decrease the number of gates needed to execute algorithms. Mid-range links, implemented by shuttling spins through a multitude of quantum dots, may entangle distant qubit registers for networked computing and allow for qubit operations at dedicated locations^14,16–18^. Furthermore, such quantum buses could provide space for the integration of on-chip control electronics^1^, depending on their footprint.

The potential of shuttling-based quantum buses has stimulated research on shuttling electron charge^19–21^ and spin^15,22–29^. While nuclear spin noise prevents high-fidelity qubit operation in gallium arsenide, demonstrations of coherent transfer of individual electron spins through quantum dots are encouraging^22–26^. In silicon, qubits can be operated with high-fidelity and this has been employed to displace a spin qubit in a double quantum dot^15,27^. Networked quantum computers, however, will require integration of qubit control and shuttling through chains of quantum dots, incorporating quantum dots that have at least two neighbors.

Meanwhile, quantum dots defined in strained germanium (Ge/SiGe) heterostructures have emerged as a promising platform for hole spin qubits^30,31^. The high quality of the platform allowed for rapid development of single spin qubits^32,33^, singlet-triplet qubits^34–36^, a four qubit processor^2^, and a 4 × 4 quantum dot array with shared gate control^11^. While the strong spin-orbit interaction allows for fast and all-electrical control, the resulting anisotropic *g*-tensor^31,37^ complicates the spin dynamics and may challenge the feasibility of a quantum bus.

Here, we demonstrate that spin qubits can be shuttled through quantum dots. These experiments are performed with two hole spin qubits in a 2 × 2 germanium quantum dot array. Importantly, we operate in a regime where we can implement single-qubit logic and coherently transfer spin qubits through an intermediate quantum dot. Furthermore, by performing experiments with precise voltage pulses and sub-nanosecond time resolution, we can mitigate finite qubit rotations induced by spin-orbit interactions. In these optimized sequences we find that the shuttling performance is limited by dephasing and can be extended through dynamical decoupling.

## Results

### Coherent shuttling of single hole spin qubits

**Fig. 1:**
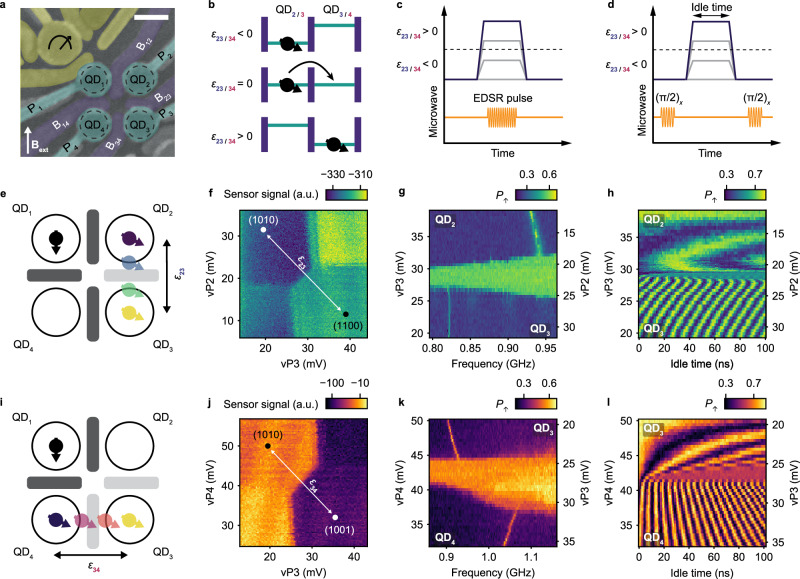
Coherent shuttling of hole spin qubits in germanium double quantum dots. **a** False colored scanning electron microscope image of a representative quantum dot (QD) device. The scale bar corresponds to 100 nm. Unless specified otherwise, an external magnetic field of 0.25 T is applied in the direction indicated by the arrow. **b** Schematic showing the principle of bucket-brigade-mode shuttling. The detuning energy *ϵ*_23/34_ between the two quantum dots is progressively changed such that it becomes energetically favorable for the hole to tunnel from one quantum dot to another. **c** Pulse sequence for experiments shown in **g** and **k**. EDSR stands for Electric Dipole Spin Resonance. **d** Pulse sequence for coherent shuttling shown in **h** and **l**. **e**, **i** Schematics illustrating the direction of spin qubit shuttling investigated in **f**–**h** and **j**–**l** respectively. Charge stability diagrams of QD_2_-QD_3_ (**f**) and QD_3_-QD_4_ (**j**). To shuttle the qubit from one site to another, the virtual plunger gate voltages are varied along the detuning axis (white arrow), which crosses the interdot charge transition line. The labels (*N*_1_*N*_2_*N*_3_*N*_4_) represent the charge occupation in the quantum dots. Probing of the resonance frequency along the detuning axis for the double quantum dot QD_2_-QD_3_ (**g**) and QD_3_-QD_4_ (**k**). *P*_*↑*_ (the probability of measuring the qubit in the $\left\vert \uparrow \right\rangle$ state) is obtained at the end of the pulse sequence. The duration of the microwave pulse is 4 μs. The ramp time used to change the detuning is 40 ns for the measurement shown in **g** and 12 ns for the measurement shown in **k**. Nearby the charge transition, the resonance frequency cannot be resolved due to a combination of effects discussed in Supplementary Note 1. Shuttling of superposition states between QD_2_-QD_3_ (**h**) and QD_3_-QD_4_ (**l**). The ramp time used to change the detuning is 40 ns for the measurement shown in **h** and 4 ns for the measurement shown in **l**.

Figure 1a shows a germanium 2 × 2 quantum dot array identical to the one used in the experiment^2^. The chemical potentials and the tunnel couplings of the quantum dots are controlled with virtual gates (vP_i_, vB_ij_), which consist of combinations of voltages on the plunger gates and the barrier gates. We operate the device with two spin qubits in quantum dots QD_1_ and QD_2_ initialized in the $\left\vert \downarrow \downarrow \right\rangle$ state (see Methods). We use the qubit in QD_1_ as an ancilla to readout the hole spin in QD_2_, using latched Pauli spin blockade^2,38,39^. The other qubit starts in QD_2_ and is shuttled to the other quantum dots by changing the detuning energies (*ϵ*_23/34_) between the quantum dots (Fig. 1b, e, i). The detuning energies are varied by pulsing the plunger gate voltages as illustrated in Fig. 1f, j. Additionally, we increase the tunnel couplings between QD_2_-QD_3_ and QD_3_-QD_4_ before shuttling to allow for adiabatic charge transfer. The hole carrying the spin remains in its orbital ground state and, with increasing ∣*ϵ*∣, the charge becomes localized in the quantum dot with the lowest chemical potential as displayed in Fig. 1b. In our experiments, we tune to have adiabatic evolution with respect to charge, and study adiabatic and diabatic shuttling with respect to spin.

The *g*-tensor of hole spin qubits in germanium is sensitive to the local electric field. Therefore, the Larmor frequency *f*_L_ is different in each quantum dot^32–34^. We exploit this effect to confirm the shuttling of a hole spin from one quantum dot to another. In Fig. 1c. we show the experimental sequence used to measure the qubit resonance frequency, while changing the detuning to transfer the qubit. Figure 1g (k) shows the experimental results for spin transfers from QD_2_ to QD_3_ (QD_3_ to QD_4_). Two regions can be clearly distinguished in between which *f*_L_ varies by 110 (130) MHz. This obvious change in *f*_L_ clearly shows that the hole is shuttled from QD_2_ to QD_3_ (QD_3_ to QD_4_) when applying a sufficiently large detuning pulse. To investigate whether such transfer is coherent, we probe the free evolution of qubits prepared in a superposition state after applying a detuning pulse (Fig. 1d)^27^. The resulting coherent oscillations are shown in Fig. 1h (l). They are visible over the full range of voltages spanned by the experiment and arise from a phase accumulation during the idle time. Their frequency *f*_osc_ is determined by the difference in resonance frequency between the starting and the end points in detuning as shown in Supplementary Fig. 1. The abrupt change in *f*_osc_ marks the point where the voltage pulse is sufficiently large to transfer the qubit from QD_2_ to QD_3_ (QD_3_ to QD_4_). These results clearly demonstrate that single hole spin qubits can be coherently transferred.

### The effect of strong spin-orbit interaction on spin shuttling

**Fig. 2:**
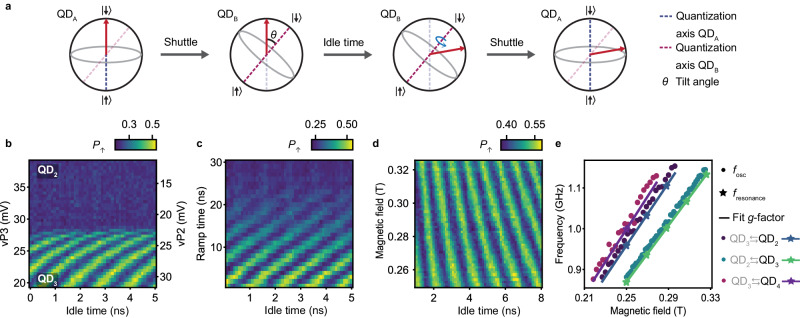
Rotations induced while shuttling by the difference in quantization axes. **a** Schematic explaining the effect of the change in quantization axis direction that the qubit experiences during the shuttling process. The difference in quantization axis between quantum dots (QDs) is caused by the strong spin-orbit interaction. **b** Oscillations in spin-up probabilities *P*_*↑*_ induced by the change in quantization axis while shuttling diabatically a qubit in a $\left\vert \downarrow \right\rangle$ state between QD_2_ and QD_3_. Ramp times of 4 ns are used for the detuning pulses. Note that the oscillations have a reduced visibility, meaning that the difference in quantization axes does not induce a full spin flip. The angle between the quantization axes of the two quantum dots can be estimated from the amplitude of the oscillations, see Supplementary Note 2A. **c** Oscillations due to the change in quantization axis at a fixed point in detuning, as function of the voltage pulse ramp time used to shuttle the spin. When the ramp time is long enough, typically above 30 ns, the spin is shuttled adiabatically and the oscillations vanish. **d** Magnetic-field dependence of the oscillations induced by the difference in quantization axis. **e** Frequency of the oscillations *f*_osc_ induced by the change in quantization axis as a function of magnetic field for different shuttling processes. The oscillation frequency *f*_osc_ for QD_3_ is extracted from measurements displayed in **d** (and similar experiments for the other quantum dot pairs) and is plotted with points. *f*_osc_ scales linearly with the magnetic field. Comparing *f*_osc_ with resonance frequencies *f*_resonance_ measured using microwave pulses (data points depicted with stars) reveals that *f*_osc_ is given by the Larmor frequency of the quantum dot towards which the qubit is shuttled (black label).

The strong spin-orbit interaction in our system has a significant impact on the spin dynamics during the shuttling. It appears when shuttling a qubit in a $\left\vert \downarrow \right\rangle$ state between QD_2_ and QD_3_ using fast detuning pulses with voltage ramps of 4 ns. Doing this generates coherent oscillations shown in Fig. 2b that appear only when the qubit is in QD_3_. They result from the strong spin-orbit interaction and the use of an almost in-plane magnetic field^40^. In this configuration, the direction of the spin quantization axis depends strongly on the local electric field^35,37,41–43^ and can change significantly between neighboring quantum dots. Therefore, rapid shuttling of a hole results in a change of angle between the spin state and the local spin quantization axis. In particular, a qubit in a basis state in QD_2_ becomes a qubit in a superposition state in QD_3_ when it is shuttled diabatically with respect to the change in quantization axis. Consequently, the spin precesses around the quantization axis of QD_3_ until it is shuttled back (Fig. 2a). This leads to qubit rotations and the aforementioned oscillations.

While these oscillations are clearly visible for voltage pulses with ramp times *t*_ramp_ of few nanoseconds, they fade as the ramp times are increased, as shown in Fig. 2c, and vanish for *t*_ramp_ > 30 ns. The qubit is then transferred adiabatically, can follow the change in quantization axis and therefore remains in the spin basis state in both quantum dots. From the visibility of the oscillations, we estimate that the quantization axis of QD_3_ (QD_4_) is tilted by at least 42^∘^ (33^∘^) compared to the quantization axis of QD_2_ (QD_3_). These values are corroborated by independent estimations made by fitting the evolution of *f*_L_ along the detuning axes (see Supplementary Note 2).

Figure 2d, e displays the magnetic field dependence of the oscillations generated by diabatic shuttling. Their frequencies *f*_osc_ increase linearly with the field and match the Larmor frequencies *f*_L_ measured for a spin in the target quantum dot. This is consistent with the explanation that the oscillations are due to the spin precessing around the quantization axis of the second quantum dot.

### Shuttling performance

**Fig. 3:**
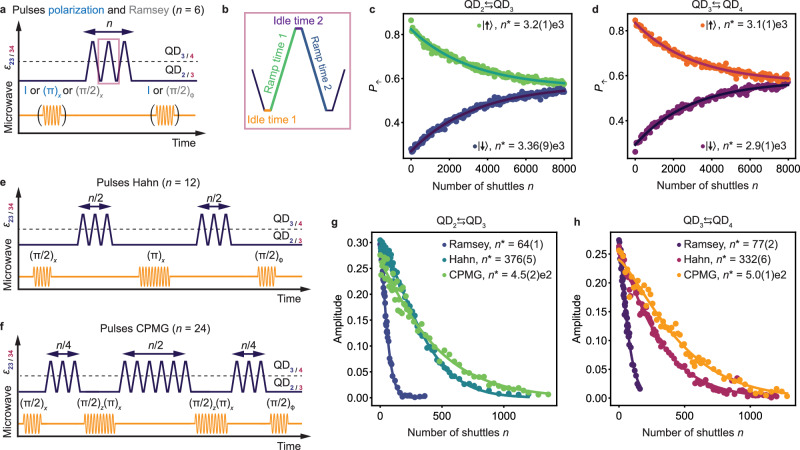
Quantifying the performance for the shuttling in double quantum dots. **a** Schematic of the pulse sequence used for quantifying the performance of shuttling basis states (blue) or a superposition state (gray). The spin qubit is prepared in the quantum dot (QD) where the shuttling experiment starts, by either applying an identity gate (shuttling a $\left\vert \downarrow \right\rangle$ state), a (*π*)_*x*_ pulse (shuttling a $\left\vert \uparrow \right\rangle$ state) or (*π*/2)_*x*_ pulse (shuttling a superposition state referred as Ramsey shuttling experiments). Detuning pulses are applied to the plunger gates to shuttle the hole from one QD to another, back and forth, and finally the appropriate pulses are applied to prepare for readout. Moving the qubit from one QD to another is counted as one shuttle *n* = 1. Since the hole is always shuttled back for readout, *n* is always even. The schematic shows an example for *n* = 6. **b** Zoom-in on the detuning pulses used for the shuttling. To make an integer number of 2*π* rotation(s) around the quantization axis of the second QD, all ramp and idle times in the pulse need to be optimized. Spin-up probabilities *P*_*↑*_ measured after shuttling *n* times a qubit prepared in a spin basis state between QD_2_ and QD_3_ (**c**) and between QD_3_ and QD_4_ (**d**). The decays of *P*_*↑*_ are fitted (solid lines) to an exponential function ${P}_{\uparrow }={P}_{0}\exp (-n/{n}^{*})+{P}_{{{{{{{{\rm{sat}}}}}}}}}$. **e** Pulse sequence used for implementing a Hahn echo shuttling experiment. In the middle of the shuttling experiment, an echo pulse (*π*)_*x*_ is applied in the QD where the spin qubit was initially prepared. Example for *n* = 12. **f** Pulse sequence for a CPMG shuttling experiment. Two (π/2)_*z*_(π)_*x*_ pulses are inserted between the shuttling pulses. Example for *n* = 24. Performance of the shuttling of superposition state between QD_2_ and QD_3_ (**g**) and QD_3_ and QD_4_ (**h**) for different shuttling sequences. The decays of the coherent amplitude *A* of the superposition state are fitted (solid lines) by ${A}_{0}\exp (-{(n/{n}^{*})}^{\alpha })$ where *α* is a fitting parameter. The uncertainties indicate one standard deviation from the best fits.

To quantify the performance of shuttling a spin qubit, we implement the experiments depicted in Fig. 3a, e, f^15,27^ and study how the state of a qubit evolves depending on the number of subsequent shuttling events. For hole spins in germanium, it is important to account for rotations induced by the spin-orbit interaction. This can be done by aiming to avoid unintended rotations, or by developing methods to correct them. An example of the first approach is transferring the spin qubits adiabatically. This implies using voltage pulses with ramps of tens of nanoseconds, which are significant with respect to the dephasing time. However, this strongly limits the shuttling performance (see Supplementary Fig. 9). Instead, we can mitigate rotations by carefully tuning the duration of the voltage pulses, such that the qubit performs an integer number of 2*π* rotations around the quantization axis of the respective quantum dot. This approach is demanding, as it involves careful optimization of the idle times in each quantum dot as well as the ramp times, as depicted in Fig. 3b. However, it allows for fast shuttling, with ramp times of typically 4 ns and idle times of 1 ns, significantly reducing the dephasing experienced by the qubit during the shuttling. We employ this strategy in the rest of our experiments.

We first characterize the shuttling of a spin qubit initialized in a basis state. We do this by preparing a qubit in a $\left\vert \uparrow \right\rangle$ or $\left\vert \downarrow \right\rangle$ state and transferring it multiple times between the quantum dots. Figure 3c, d displays the spin-up fraction *P*_*↑*_ measured as a function of the number of shuttling steps *n*. The probability of ending up in the initial state shows a clear exponential dependence on *n*. No oscillations of *P*_*↑*_ with *n* are visible, confirming that the pulses have been successfully optimized to account for unwanted spin rotations. We extract the characteristic decay constants n^*^ by fitting the data for the shuttling of qubits prepared in $\left\vert \uparrow \right\rangle$ and $\left\vert \downarrow \right\rangle$ states separately as they originate from distinct sets of experiments. In all cases, we find a characteristic decay n^*^ ≃ 3000 shuttles between quantum dots, corresponding to a polarization transfer fidelities of $F=\exp (-1/{n}^{*})\simeq 99.97$ % per shuttle within the sequence. This is similar to the fidelities reached in silicon devices^15,27^, despite the anisotropic *g*-tensors due to the strong spin-orbit interaction in our platform.

The exponential decay of the spin polarization to approximately 0.5 can emerge from different effects. At the charge anticrossing, the spin polarization life time is strongly reduced (see Supplementary Fig. 3), due to high frequency charge noise and coupling to phonons^44^. Passing the charge anticrossing repeatedly thus leads to a randomization of the spin. Moreover, while the qubit starts in a basis state, it undergoes coherent rotations due to the diabatic spin shuttling and it is in a superposition state in the second quantum dot. The qubit, although initially in a spin basis state, then becomes sensitive to dephasing which can also lead to an exponential decay of *P*_*↑*_. The experimental decay observed probably results from a combination of these mechanisms.

We emphasize that the exact impact of dephasing on the performance of the shuttling of spin basis state depends on the difference in quantization axes of the quantum dots and on the pulse sequence used (see Supplementary Note 8). In our experiment, the dephasing is probably mitigated by a decoupling effect induced by repeatedly waiting in the initial quantum dot (see explanations Supplementary Note 8). While extrapolating this result to a long chain of quantum dots is not straightforward, similar noise-averaging effects may occur in the presence of spatially correlated noise in the chain^45^. In the absence of decoupling effects and for the purpose of shuttling basis states, adiabatic shuttling still provides a good alternative as we find n^*^ to remain above 1000, corresponding to fidelities per shuttle within the sequence above 99.90% (see Supplementary Fig. 9).

We now focus on the performance of coherent shuttling. We prepare a superposition state via an EDSR (*π*/2)_*x*_ pulse, shuttle the qubit, apply another *π*/2 pulse and measure the spin state. Importantly, one must account for $\hat{z}$-rotations experienced by the qubits during the experiments and the corresponding phase accumulation defined with respect to the qubit rotating frame in the initial quantum dots. The latter can be equivalently defined with respect to the lab frame. Therefore, we vary the phase of the EDSR pulse ϕ for the second *π*/2 pulse i.e. the final pulse is a (ϕ)_*z*_(π/2)_*x*_ = (π/2)_ϕ_ pulse. For each *n*, we then extract the amplitude *A* of the *P*_*↑*_ oscillations that appear as function of ϕ^15,27^. Figure 3g, h shows the evolution of *A* as a function of *n* for shuttling between adjacent quantum dots. We fit the experimental results using ${A}_{0}\exp (-{(n/{n}^{*})}^{\alpha })$ and find characteristic decay constants ${n}_{23}^{*}=64\pm 1$ and ${n}_{34}^{*}=77\pm 2$. Remarkably, these numbers compare favorably to n^*^ ≃ 50 measured in a SiMOS electron double quantum dot^27^, where the spin-orbit coupling is weak.

The exponents, *α*_23_ = 1.36 ± 0.05 and *α*_34_ = 1.28 ± 0.06, characterize the spectrum of the noise experienced by the qubit while it is shuttled and suggest that the noise is neither purely quasistatic nor white. The non-integer values of *α* contrast with observations in silicon^15,27^, and suggest that the shuttling of hole spins in germanium is limited by other mechanisms. Two types of errors can be distinguished. Errors may occur during the diabatic part of the spin dynamics. On the other hand, errors can also be induced by the dephasing experienced by the qubits during the finite time spent in each quantum dot, including the ramp times (see Supplementary Note 8). To investigate the effect of dephasing, we modify the shuttling sequence and include a (*π*)_*x*_ echoing pulse in the middle as displayed in Fig. 3e. We note that the echoing pulses are defined with respect to the rotating frame of the qubit in the starting quantum dots. Figure 3g, h shows the experimental results and it is clear that in germanium the coherent shuttling performance is improved significantly using an echo pulse: we can extend the shuttling by a factor of four to five, reaching a characteristic decay of more than 300 shuttles. Similarly, the use of CPMG sequences incorporating two decoupling (π/2)_*z*_(π)_*x*_ pulses (Fig. 3f) allows further, although modest, improvements. These enhancements in the shuttling performance confirm that dephasing is limiting the shuttling performance, contrary to observations in SiMOS^27^. We speculate that the origin of the difference is two-fold. Firstly, due to the stronger spin-orbit interaction, the spin is more sensitive to charge noise, resulting in shorter dephasing times. Secondly, the excellent control over the potential landscape in germanium allows minimizing the errors which are due to the shuttling itself.

While the results obtained for the diabatic shuttling in germanium double quantum dots are similar to those attained in silicon devices for adiabatic shuttling^15,27^, one should be careful in comparing and extrapolating them to predict the performance of shuttling through longer quantum dot chains. Quantum dot chains that would allow to couple spin qubits over appreciable length scales will put higher demands on tuning, on uniformity, and the ability to tune all couplings. Moreover, a qubit shuttled through a chain may probe different noise environments which can further affect the performance.

### Shuttling through intermediate quantum dots

For distant qubit coupling, it is essential that a qubit can be coherently shuttled through chains of quantum dots. This is more challenging, as it requires control and optimization of a larger amount of parameters while more noise sources may couple to the system. Within a chain, a quantum dot will have at least two neighbors. To transport spin states from one site to another they have to pass through intermediate quantum dots. Therefore, an array of three quantum dots could be considered as the minimum size to explore the performance of shuttling in a chain.

**Fig. 4:**
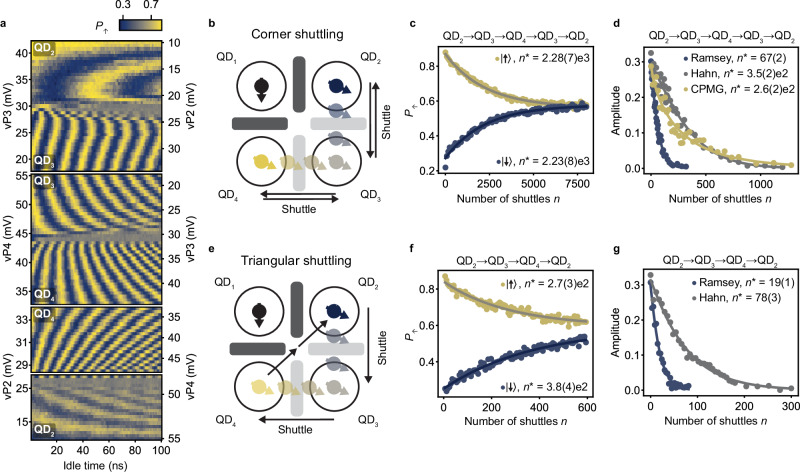
Coherent shuttling through quantum dots. **a** Results of free evolution experiments while shuttling through quantum dots (QDs), similar to those displayed in Fig. 1h, l for the corner and triangular shuttling processes. In these experiments, the amplitude of the detuning pulse is increased in steps, in order to shuttle a qubit from QD_2_ to QD_3_ and back (top panel), from QD_2_ to QD_3_ to QD_4_ and back (second panel). The measurement in the third panel is identical to the measurement in the second panel, but the final point in the charge stability diagram is stepped towards the charge degeneracy point between QD_2_ and QD_4_. In the bottom panel the qubit is shuttled in a triangular fashion: from QD_2_ to QD_3_ to QD_4_ to QD_2_. The ramp times for this experiment are chosen in such a way that the shuttling is adiabatic with respect to the changes in quantization axis. Schematic illustrating the shuttling of a spin qubit around the corner: from QD_2_ to QD_3_ to QD_4_ and back via QD_3_ (**b**) and in a triangular fashion: from QD_2_ to QD_3_ to QD_4_ and directly back to QD_2_ (**e**). The double arrow from QD_4_ to QD_2_ indicates that this pulse is made in two steps, in order for the spin to shuttle via the charge degeneracy point of QD_4_ - QD_2_ and avoid crossing charge transition lines. Performance for the corner shuttling (**c**) and the triangular shuttling (**f**) of a qubit prepared in the basis states. The decays of the spin-up probabilities *P*_*↑*_ are fitted (solid lines) by ${P}_{0}\exp (-n/{n}^{*})+{P}_{{{{{{{{\rm{sat}}}}}}}}}$. Performance for shuttling a qubit prepared in a superposition state for the corner shuttling (**d**) and the triangular shuttling (**g**) and for different shuttling sequences. The decays of the coherent amplitude *A* are fitted (solid lines) by ${A}_{0}\exp (-{(n/{n}^{*})}^{\alpha })$. Shuttling performance for different processes are summarized in Supplementary Table 1. The uncertainties indicate one standard deviation from the best fits.

We perform two types of experiments to probe the shuttling through chains of quantum dots, labeled corner shuttling and triangular shuttling. Figure 4b shows a schematic of the corner shuttling, which consists of transferring a qubit from QD_2_ to QD_3_ to QD_4_ and back along the same route. The triangular shuttling, depicted in Fig. 4e, consists of shuttling the qubit from QD_2_ to QD_3_ to QD_4_, and then directly back to QD_2_, without passing through QD_3_ (for the charge stability diagram QD_4_-QD_2_ and a detailed description see Supplementary Note 5).

To probe the feasibility of shuttling through a quantum dot, we first measure the free evolution of a superposition state while varying the detuning between the respective quantum dots. The results are shown in Fig. 4a. We find a remarkably clear coherent evolution for hole spin transfer from QD_2_ to QD_3_ to QD_4_ and to QD_2_. We observe one sharp change in the oscillation frequency for each transfer to the next quantum dot. We also note that after completing one round of the triangular shuttling, the phase evolution becomes constant, in agreement with a qubit returning to its original position. We thereby conclude that we can shuttle through quantum dots as desired.

We now focus on quantifying the performance of shuttling through quantum dots by repeated shuttling experiments. To allow comparisons with previous experiments, we define *n* as the number of shuttling steps between two quantum dots. Meaning that one cycle in the corner shuttling experiments results in *n* = 4, while a loop in the triangular shuttling takes *n* = 3 steps. The results for shuttling basis states are shown in Fig. 4c, f. We note that the spin polarization decays faster compared to the shuttling in double quantum dots, in particular for the triangular shuttling. The corresponding fidelities per shuttle within the sequence are *F* ≃ 99.96% for the corner shuttling and *F* ≥ 99.63% for the triangular shuttling.

For the corner shuttling, the faster decay of the basis states suggests a slight increase of the systematic error per shuttle. This may originate from the use of a more elaborated pulse sequence, which makes pulse optimization more challenging. Nonetheless, the characteristic decay constant n^*^ remains above 2000 and corresponds to effective distances beyond 300 *μ*m (taking a 140 nm quantum dot spacing). The fast decay for the triangular shuttling is likely originating from the diagonal shuttling step. The tunnel coupling between QD_2_ and QD_4_ is low and more challenging to control, due to the absence of a dedicated barrier gate. The low tunnel coupling demands slower ramp times (*t*_ramp_ ≃ 36 ns) for the hole transfer. This increases the dephasing experienced by the qubit during each shuttle and also the time spent close to the (1100)-(1001) charge degeneracy point, where fast spin randomization will likely occur.

Remarkably, we find that the performance achieved for the coherent corner shuttling (as shown in Fig. 4d) are comparable to those of coherent shuttling between neighboring quantum dots. This stems from the performance being limited by dephasing. However, the performance for the CPMG sequence appears inferior when compared to the single echo-pulse sequence. Since the shuttling sequence becomes more complex, we speculate that it is harder to exactly compensate for the change in quantization axes. Imperfect compensation may introduce errors, which are  not fully decoupled using the CPMG sequence. Alternatively, simulations shown in Supplementary Fig. 14 suggest that the decoupling achieved using a CPMG sequence depends on the idle time in the initial quantum dots. For an idle time corresponding to a (2*k* + 1)*π* (with *k* an integer) phase accumulation, the decoupling achieved using either an ideal echo or a CPMG sequence is very similar. In such a scenario, the effect of imperfect decoupling pulses would become more apparent in a CMPG sequence and would lead to decreased performance.

The performance of the coherent triangular shuttling, displayed in Fig. 4g, fall short compared to the corner shuttling. Yet, the number of shuttles reached remains limited by dephasing as shown by the large improvement of n^*^ obtained using dynamical decoupling. The weaker performance are thus predominantly a consequence of the use of longer voltage ramps. A larger number of coherent shuttling steps may be achieved by increasing the diagonal tunnel coupling, which could be obtained by incorporating dedicated barrier gates.

## Discussion

We have demonstrated coherent spin qubit shuttling through quantum dots. While holes in germanium provide challenges due to an anisotropic *g*-tensor, we find that spin basis states can be shuttled n^*^ = 2230 times and coherent states up to n^*^ = 67 times and even up to n^*^ = 350 times when using echo pulses. The small effective mass and high uniformity of strained germanium allow for a comparatively large quantum dot spacing of 140 nm. This results in effective length scales for shuttling basis states of *l*_spin_ = 312 μm and for coherent shuttling of *l*_coh_ = 9 μm. By including echo pulses we can extend the effective length scale to *l*_coh_ = 49 μm. These results compare favorably to effective lengths obtained in silicon^15,27–29^. However, we note that, in general, extrapolating the performance of shuttling experiments over few sites to predict the performance of practical shuttling links requires caution. Quantum dot chains that would allow to couple spin qubits over appreciable length scales will put higher demands on tuning, uniformity, and the ability to tune all the couplings, making the optimization of the shuttling more challenging. Moreover, the spin dynamics and thus the coherent shuttling performance will depend on the noise in the quantum dot chain. For example, if the noise is local, echo pulses may prove less effective. However, in that case, motional narrowing^22,25,29,45–47^ may facilitate the shuttling.

Furthermore, operating at even lower magnetic fields will boost the coherence times^4,37^ and thereby increase the shuttling performance. Moreover, at lower magnetic fields the Larmor frequency is lower, which eases the requirements for the precision of the timing of the shuttling pulses. At very low fields, charge noise might not be the limiting noise source anymore and even further improvements may be achieved exploiting purified germanium^4,37,40^. Finally, shuttling could help mitigate problems in qubit addressability which may arise at low magnetic field.

While we have focused on bucket-brigade-mode shuttling, our results also open the path to conveyor-mode shuttling in germanium, where qubits would be coherently displaced in propagating potential wells using shared gate electrodes. This complementary approach holds promise for making scalable mid-range quantum links and has recently been successfully investigated in silicon^29^, though on limited length scales. For holes in germanium, the small effective mass and absence of valley degeneracy will be beneficial in conveyor-mode shuttling. Rotations induced by the spin-orbit interaction while shuttling in conveyor-mode could be compensated by applying an appropriate EDSR pulse after the qubit transfer. Such methods could also be used in bucket-brigade-mode shuttling, as suggested by preliminary experiments shown in Supplementary Note 9. It may allow for even faster qubit transfers and thus shuttling over longer distances.

Importantly, quantum links based on shuttling and spin qubits are realized using the same manufacturing techniques. Their integration in quantum circuits may provide a path toward networked quantum computing.

## Methods

### Materials and device fabrication

The device is fabricated on a strained Ge/SiGe heterostructure grown by chemical vapor deposition^30,48^. From bottom to top the heterostructure is composed of a 1.6-μm thick relaxed Ge layer, a 1-μm step graded Si_1−*x*_Ge_*x*_ (*x* going from 1 to 0.8) layer, a 500 nm relaxed Si_0.2_Ge_0.8_ layer, a strained 16 nm Ge quantum well, a 55 nm Si_0.2_Ge_0.8_ spacer layer and a < 1-nm thick Si cap. Contacts to the quantum well are made by depositing 30 nm of aluminum on the heterostructure after etching of the oxidized Si cap. The contacts are isolated from the gate electrodes using a 7 nm aluminum oxide layer deposited by atomic layer deposition. The gates are defined by depositing Ti/Pd bilayers. They are separated from each other by 7 nm of aluminum oxide.

### Experimental procedure

To perform the experiments presented, we follow a systematic procedure composed of several steps. We start by preparing the system in a (1,1,1,1) charge state with the hole spins in QD_1_ and QD_2_ initialized in a $\left\vert \downarrow \right\rangle$ state, while the other spins are randomly initialized. Subsequently, QD_3_ and QD_4_ are depleted to bring the system in a (1,1,0,0) charge configuration. After that, the virtual barrier gate voltage vB_12_ is increased to isolate the ancilla qubit in QD_1_. The tunnel couplings between QD_2_ and QD_3_ and, depending on the experiment, between QD_3_ and QD_4_ are then increased by lowering the corresponding barrier gate voltages on vB_23_ and vB_34_. This concludes the system initialization.

Thereafter, the shuttling experiments are performed. In the shuttling experiments, waiting times up to 10 ns are included on both sides of each microwave pulse. These waiting times are short compared to the microwave pulse times as well as the qubit coherence times. Note that to probe the shuttling between QD_3_ and QD_4_, the qubit is first transferred adiabatically (with respect to the change in quantization axis) from QD_2_ to QD_3_. To determine the final spin state after the shuttling, the qubit is transferred back adiabatically to QD_2_. Next, the system is brought back in the (1,1,1,1) charge state, the charge regime in which the readout is optimized. This is done by first increasing vB_23_ and vB_34_, then decreasing vB_12_ and finally reloading one hole in both QD_3_ and QD_4_. We finally readout the spin state via latched Pauli spin blockade by transferring the qubit in QD_1_ to QD_2_ and integrating the signal from the charge sensor for 7 μs. Spin-up probabilities are determined by repeating each experiment a few thousand times. Details about the experimental setup can be found in ref. ^2^.

### Achieving sub-nanosecond resolution on the voltage pulses

For these experiments, we use voltage pulses applied to the electrostatic gates by the arbitrary wave form generators (AWGs). These pulses are compiled as a sequence of ramps, using a control software. The ramps are defined by high precision floating points: time stamps and voltages. The maximum resolution in time is set by the maximum sample rate of the AWGs, which is 1 GSa/s and which translates to a resolution of 1 ns. Using this sample rate, the signal that is outputted by the AWGs has discrete steps, as depicted in Supplementary Fig. 2a. Simply moving this sampled pulse in time is only possible with a precision of 1 ns. However, it is possible to achieve sub-nanosecond resolution by slightly adjusting the voltages of the pulse instead. As illustrated in Supplementary Fig. 2a, in this way it is possible to delay a pulse with less than 1 ns. Quantitatively: to achieve a time delay of *τ*, the voltages forming the ramp are shifted by $-\tau \frac{\,{{\mbox{d}}}\,{V}_{{{{{{{{\rm{ramp}}}}}}}}}(t)}{\,{{\mbox{d}}}\,t}$. The output of the AWGs has a higher order low-pass filter with a cut-off frequency of approximately 400 MHz. This filter smoothens the output signal and effectively removes the effect of the time discretization, as is shown in Supplementary Fig. 2b. The time shift of the pulse is not affected by the filter, since it does not change the frequency spectrum of the pulse. To summarize, combining the high precision in the voltages of the pulse with the output filtering of the AWGs allows to output a smooth voltage ramp that is delayed by *τ* < 1 ns, despite the limited sampling rate. Applying this technique to all voltage ramps results in sub-nanosecond resolution on the overall pulse sequence.

### Supplementary information

Supplementary Information

Peer Review File

## Data Availability

The data generated in this study have been deposited in the Zenodo repository under accession code: https://zenodo.org/records/11203148.

## References

[1] Vandersypen LMK (2017). Interfacing spin qubits in quantum dots and donors-hot, dense, and coherent. npj Quantum Inf..

[2] Hendrickx NW (2021). A four-qubit germanium quantum processor. Nature.

[3] Philips SGJ (2022). Universal control of a six-qubit quantum processor in silicon. Nature.

[4] Lawrie WIL (2023). Simultaneous single-qubit driving of semiconductor spin qubits at the fault-tolerant threshold. Nat. Commun..

[5] Mills AR (2022). Two-qubit silicon quantum processor with operation fidelity exceeding 99%. Sci. Adv..

[6] Noiri A (2022). Fast universal quantum gate above the fault-tolerance threshold in silicon. Nature.

[7] Xue X (2022). Quantum logic with spin qubits crossing the surface code threshold. Nature.

[8] Petit L (2020). Universal quantum logic in hot silicon qubits. Nature.

[9] Yang CH (2020). Operation of a silicon quantum processor unit cell above one kelvin. Nature.

[10] Camenzind LC (2022). A hole spin qubit in a fin field-effect transistor above 4 kelvin. Nat. Electron..

[11] Borsoi F (2024). Shared control of a 16 semiconductor quantum dot crossbar array. Nat. Nanotechnol..

[12] Van Meter R, Horsman D (2013). A blueprint for building a quantum computer. Commun. ACM.

[13] Franke DP, Clarke JS, Vandersypen LMK, Veldhorst M (2019). Rent’s rule and extensibility in quantum computing. Microprocessors Microsyst..

[14] Li R (2018). A crossbar network for silicon quantum dot qubits. Sci. Adv..

[15] Noiri A (2022). A shuttling-based two-qubit logic gate for linking distant silicon quantum processors. Nat. Commun..

[16] Taylor JM (2005). Fault-tolerant architecture for quantum computation using electrically controlled semiconductor spins. Nat. Phys..

[17] Boter JM (2022). The spider-web array–a sparse spin qubit array. Phys. Rev. Appl..

[18] Künne, M. et al. The SpinBus architecture for scaling spin qubits with electron shuttling. *Nat. Commun.*** 15,** 4977 (2024). 10.1038/s41467-024-49182-4 PMC11166970 38862531

[19] Mills AR (2019). Shuttling a single charge across a one-dimensional array of silicon quantum dots. Nat. Commun..

[20] Seidler I (2022). Conveyor-mode single-electron shuttling in Si/SiGe for a scalable quantum computing architecture. npj Quantum Inf..

[21] Xue R (2024). Si/SiGe QuBus for single electron information-processing devices with memory and micron-scale connectivity function. Nat. Commun..

[22] Flentje H (2017). Coherent long-distance displacement of individual electron spins. Nat. Commun..

[23] Fujita T, Baart TA, Reichl C, Wegscheider W, Vandersypen LMK (2017). Coherent shuttle of electron-spin states. npj Quantum Inf..

[24] Mortemousque P-A (2021). Coherent control of individual electron spins in a two-dimensional quantum dot array. Nat. Nanotechnol..

[25] Mortemousque P-A (2021). Enhanced spin coherence while displacing electron in a two-dimensional array of quantum dots. PRX Quantum.

[26] Jadot B (2021). Distant spin entanglement via fast and coherent electron shuttling. Nat. Nanotechnol..

[27] Yoneda J (2021). Coherent spin qubit transport in silicon. Nat. Commun..

[28] Zwerver AMJ (2023). Shuttling an electron spin through a silicon quantum dot array. PRX Quantum.

[29] Struck T (2024). Spin-EPR-pair separation by conveyor-mode single electron shuttling in Si/SiGe. Nat. Commun..

[30] Sammak A (2019). Shallow and undoped germanium quantum wells: A playground for spin and hybrid quantum technology. Adv. Funct. Mater..

[31] Scappucci G (2021). The germanium quantum information route. Nat. Rev. Mater..

[32] Hendrickx NW (2020). A single-hole spin qubit. Nat. Commun..

[33] Hendrickx NW, Franke DP, Sammak A, Scappucci G, Veldhorst M (2020). Fast two-qubit logic with holes in germanium. Nature.

[34] Jirovec D (2021). A singlet-triplet hole spin qubit in planar Ge. Nat. Mater..

[35] Jirovec D (2022). Dynamics of hole singlet-triplet qubits with large *g*-factor differences. Phys. Rev. Lett..

[36] Wang C-A (2023). Probing resonating valence bonds on a programmable germanium quantum simulator. npj Quantum Inf..

[37] Hendrickx, N. W. et al. Sweet-spot operation of a germanium hole spin qubit with highly anisotropic noise sensitivity. *Nat. Mater.* (2024). 10.1038/s41563-024-01857-5 PMC11230914 38760518

[38] Studenikin SA (2012). Enhanced charge detection of spin qubit readout via an intermediate state. Appl. Phys. Lett..

[39] Harvey-Collard P (2018). High-fidelity single-shot readout for a spin qubit via an enhanced latching mechanism. Phys. Rev. X.

[40] Lawrie, W. I. L. *Spin qubits in silicon and germanium*. PhD Thesis, Technical Univ. Delft (2022).

[41] Mutter PM, Burkard G (2021). All-electrical control of hole singlet-triplet spin qubits at low-leakage points. Phys. Rev. B.

[42] Bosco S, Benito M, Adelsberger C, Loss D (2021). Squeezed hole spin qubits in Ge quantum dots with ultrafast gates at low power. Phys. Rev. B.

[43] Wang, C.-A., Scappucci, G., Veldhorst, M. & Russ, M. Modelling of planar germanium hole qubits in electric and magnetic fields. Preprint at https://arxiv.org/abs/2208.04795 (2022). 10.1038/s41534-024-00897-8 PMC11486654 39429902

[44] Krzywda JA, Cywiński Ł (2021). Interplay of charge noise and coupling to phonons in adiabatic electron transfer between quantum dots. Phys. Rev. B.

[45] Bosco, S., Zou, J. & Loss, D. High-fidelity spin qubit shuttling via large spin-orbit interactions. *PRX Quantum* **5**, 020353 (2024).

[46] Huang P, Hu X (2013). Spin qubit relaxation in a moving quantum dot. Phys. Rev. B.

[47] Langrock V (2023). Blueprint of a scalable spin qubit shuttle device for coherent mid-range qubit transfer in disordered Si/SiGe/SiO_2_. PRX Quantum.

[48] Lodari M (2019). Light effective hole mass in undoped Ge/SiGe quantum wells. Phys. Rev. B.

