# Supplementary material for: Coherent spin qubit shuttling through germanium quantum dots
Source: Nat Commun. 2024 Jul 8;15:5716. doi: 10.1038/s41467-024-49358-y (PMC11231167; doi:10.1038/s41467-024-49358-y)
Supplement: Supplementary file 1 — Supplementary Information [file 41467_2024_49358_MOESM1_ESM.pdf]

Supplementary Information: Coherent spin qubit shuttling through germanium quantum dots

Floor van Riggelen-Doelman,[1] Chien-An Wang,[1] Sander L. de Snoo,[1] William I. L. Lawrie,[1] Nico W. Hendrickx,[1] Maximilian Rimbach-Russ,[1] Amir Sammak,[2] Giordano Scappucci,[1] Corentin Déprez,[1] and Menno Veldhorst[1]

[1] *QuTech and Kavli Institute of Nanoscience, Delft University of Technology, PO Box 5046, 2600 GA Delft, The Netherlands*

[2] *QuTech and Netherlands Organisation for Applied Scientific Research (TNO), 2628 CK, Delft, The Netherlands*

This Supplementary Material includes:

- Supplementary Notes 1-9
- Supplementary Figures 1-15
- Supplementary Table 1-2
- Supplementary References 1-10

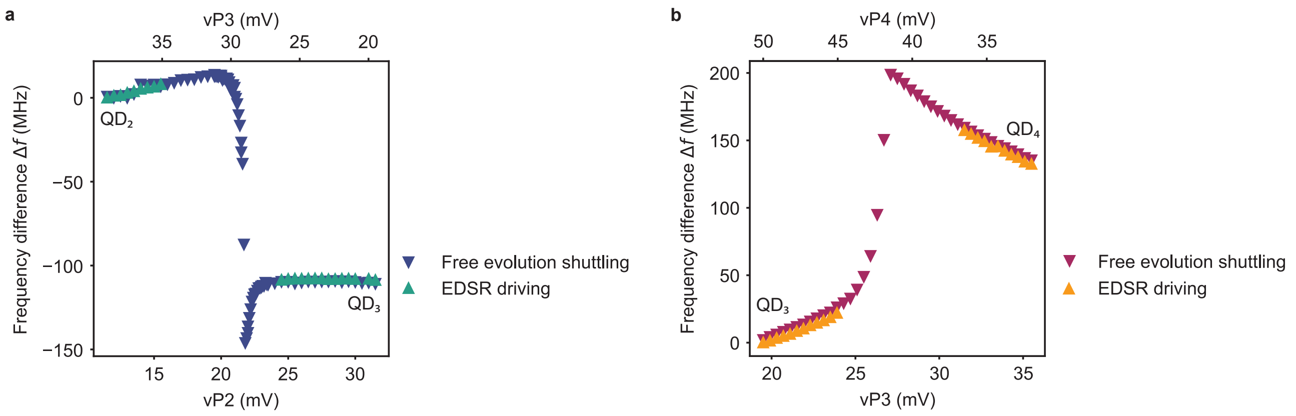

Supplementary Figure 1. **Evolution of the Larmor frequency for shuttling in double quantum dots.** **a**, **b**, Larmor frequency differences $\Delta f = f_{\rm L}({\rm vP2, vP3}) - f_{\rm L}^{\rm QD2}$ (a) and $\Delta f = f_{\rm L}({\rm vP3, vP4}) - f_{\rm L}^{\rm QD3}$ (b) measured along the detuning axis of QD_2-QD_3 (a) and QD_3-QD_4 (b). The quantum dot where the shuttling experiment starts is taken as the reference point for the frequency. Δf is independently evaluated from measurements of the resonance frequency using an EDSR pulse (data displayed in Fig. 1.g and k) and from the frequency of the coherent oscillations that appear when a qubit is shuttled in a superposition state (data displayed in Fig. 1.h and l). Both sets of data points overlap in (a) and (b), confirming that the coherent oscillations arise due to a change in Larmor frequency along the detuning axis. For the free evolution experiments, the shuttling between QD_2 and QD_3 (shown in (a)) is completely adiabatic (ramp times of 40 ns) while the shuttling between QD_3 and QD_4 (shown in (b)) is only partially adiabatic (ramp times of 4 ns). In the latter case, the frequency difference measured is barely affected by the limited adiabaticity as the visibility M of the oscillations induced by the change in quantization axes ($M < 0.1$ from Supplementary Figure 4) is sufficiently small compared to that of the oscillations arising from the phase evolution of the superposition state ($V \approx 0.5$ when the hole is in QD_4). Moreover, the Larmor frequency of spins in both QD_3 and QD_4 are close to 1 GHz. The free evolution experiments were performed with a time resolution of 1 ns, meaning that the oscillations due to the diabaticity of the shuttling only show up as an aliasing pattern and do not disturb the oscillations due to free evolution.

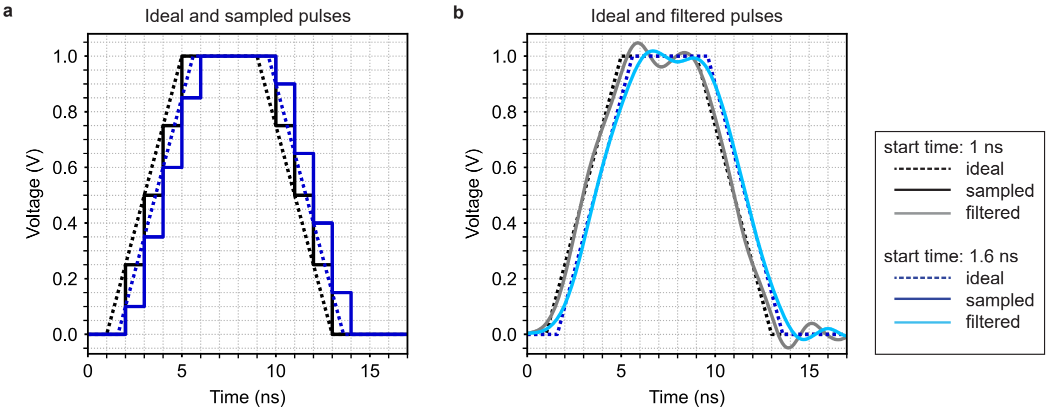

Supplementary Figure 2. **AWG pulses with sub-nanosecond precision.** **a**, Ideal voltage pulse starting at 1 ns (dotted black) and ideal voltage pulse delayed by 0.6 ns with respect to the first one (dotted blue). For both pulses, the discretized sampling is plotted in solid lines. The delay between the two pulses combined with the sampling, leads to a shift of the voltage steps. **b**, Ideal pulses (starting at 1 ns and 1.6 ns) and pulses as outputted by the AWG after filtering, without (light grey) and with the 0.6 ns delay (light blue). The filtering clearly smoothens the sampled pulses such that the outputted signals closely resemble the ideal pulses.

Shuttling process	n^* for $\|\downarrow\rangle$ transfer	n^* for $\|\uparrow\rangle$ transfer	n^* for $\frac{\|\downarrow\rangle - i\|\uparrow\rangle}{\sqrt{2}}$ transfer	α for $\frac{\|\downarrow\rangle - i\|\uparrow\rangle}{\sqrt{2}}$ transfer
$QD_2 \rightleftarrows QD_3$	$(3.36 \pm 0.09) \times 10^3$	$(3.2 \pm 0.1) \times 10^3$	Ramsey: 64 ± 1<br>Hahn: 376 ± 5<br>CPMG: $(4.5 \pm 2) \times 10^2$	Ramsey: 1.36 ± 0.05<br>Hahn: 1.44 ± 0.04<br>CPMG: 1.14 ± 0.06
$QD_3 \rightleftarrows QD_4$	$(2.9 \pm 0.1) \times 10^3$	$(3.1 \pm 0.1) \times 10^3$	Ramsey: 77 ± 2<br>Hahn: 332 ± 6<br>CPMG: $(5.0 \pm 0.1) \times 10^2$	Ramsey: 1.28 ± 0.06<br>Hahn: 1.17 ± 0.04<br>CPMG: 1.30 ± 0.07
Corner<br>$QD_2 \rightarrow QD_3 \rightarrow QD_4$<br>$\rightarrow QD_3 \rightarrow QD_2$	$(2.23 \pm 0.08) \times 10^3$	$(2.28 \pm 0.07) \times 10^3$	Ramsey: 67 ± 2<br>Hahn: $(3.5 \pm 0.2) \times 10^2$<br>CPMG: $(2.6 \pm 0.2) \times 10^2$	Ramsey: 1.11 ± 0.06<br>Hahn: 1.2 ± 0.1<br>CPMG: 0.76 ± 0.07
Triangular<br>$QD_2 \rightarrow QD_3 \rightarrow QD_4 \rightarrow QD_2$	$(3.8 \pm 0.4) \times 10^2$	$(2.7 \pm 0.3) \times 10^2$	Ramsey: 19 ± 1<br>Hahn: 78 ± 3	Ramsey: 1.08 ± 0.07<br>Hahn: 1.07 ± 0.05

Supplementary Table 1. **Summary of shuttling performance.** For the spin basis state shuttling experiments, the spin polarization decays are fitted by $P_0 \exp(-(n/n^*)) + P_{\mathrm{sat}}$, with n the number of shuttles. For the coherent shuttling experiments, the coherence decays are fitted by $A_0 \exp\left(-(n/n^*)^\alpha\right)$. n^* represents the number of shuttles that can be achieved before the polarization or the coherence drops by $1/e$. The uncertainties indicate one standard deviation from the best fits.

Supplementary Note 1. Spin randomization nearby the interdot charge transition

In Fig. 1.g and k, we show the evolution of the qubit resonance frequency f_{L} along the detuning axis of the QD_2-QD_3 quantum dot pair and of the QD_3-QD_4 quantum dot pair. f_{L} is measured by shuttling the spin and applying a 4 µs long EDSR pulse on one plunger gate. While f_{L} can be clearly determined when the hole is well-localized in one quantum dot, it cannot be measured nearby the charge transition as the spin-up probability has a high value over the whole range of frequency spanned. We think that this is the result of a combination of different effects.

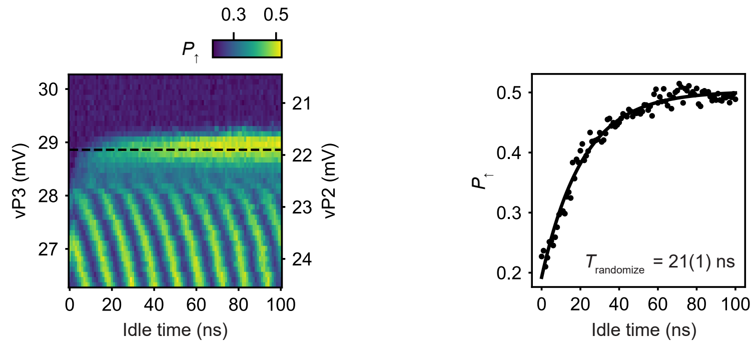

Supplementary Figure 3. **Randomization of spin states around the QD_2 - QD_3 charge transition.** **a**, Shown is the result of the shuttling of a spin in the $|\downarrow\rangle$ state while changing the detuning and the idle time. Note that in contrast to the measurement shown in Fig. 2.a of the main text, the time resolution on the x-axis is not large enough to distinguish the oscillations with gigahertz frequency, instead what is visible here is an aliasing pattern. **b**, The data plotted and fitted here correspond to a linecut of the data in (a), taken along the dashed line. The typical time it takes for the spin to randomize (while starting in the $|\downarrow\rangle$ state) is 21 ± 1 ns and is extracted by fitting an exponential function. The uncertainty corresponds to one standard deviation from the best fit.

Since the two quantum dots have different quantization axes, the system effectively behaves as a flopping-mode qubit nearby the charge transition [1–4] and the EDSR driving is thus expected to be more efficient. This appears, in Fig. 1.g, when the qubit is in QD_2: along the resonance line, we observe an alternation of high and low spin-up probabilities that witness rapid variations of the Rabi frequency. As a consequence, the power broadening increases significantly in the vicinity of the charge transition which prevents us from resolving the qubit resonance frequency. We have not observed such effects in the other quantum dots and speculate this is due to the driving efficiency and the depahsing. In this studied device, the four qubits can usually be efficiently driven with one particular plunger

gate. This drivability is likely the origin why the effect disappears once the qubit is in QD_3 in Fig. 1g.

The gradient of shear strains induced by the thermal contraction of the gate electrodes can lead to large increases of the Rabi frequency [5]. It is likely that this effect is enhanced in the vicinity of the charge transition, as the hole is delocalized between the two quantum dots and its wavefunction extends below the edges of several gates. Finally, nearby the charge transition, excitations to higher energy states induced by charge noise are more likely to occur [6], especially on the relatively long timescale of 4 µs. These transitions to higher energy states lead to a randomization of the spin states, which explains the large spin-up probabilities observed over the full frequency range.

This last argument is supported by the data shown in Supplementary Figure 3. This figure shows the result of the shuttling of a qubit in a $|\downarrow\rangle$ state while changing the detuning and varying the idle time (similar to Fig. 2.a of the main text). It becomes clear that once the spin approaches the charge anticrossing between QD_2 and QD_3 (indicated by the striped black line in Supplementary Figure 3.a), the time it takes for the spin state to be randomized decreases very rapidly to about $T_{\mathrm{randomize}} = 21 \pm 1$ ns (fit to an exponential decay shown in Supplementary Figure 3.b). The randomization of the spin close to the charge anticrossing could also be an explanation for the fact that the spin-up probability measured for shuttling basis states decays to the value of 0.5 instead of 0.

Supplementary Note 2. Quantifying the quantization axis tilt angle

A. Estimation based on the visibility of the oscillations induced by the change in quantization axis

The tilt angle θ between the quantization axis of two different quantum dots can be estimated based on the amplitude of the oscillations induced by diabatically shuttling a qubit in the $|\downarrow\rangle$ state. This approximation relies on a simple geometric construction in the Bloch sphere.

Supplementary Figure 4.a shows the Bloch sphere projected on the plane defined by the quantization axes of the two quantum dots (dark blue and pink). At the beginning of the experiment, the qubit is initialized in the $|\downarrow\rangle$ state (red arrow). After shuttling to the neighboring quantum dot, the qubit state changes due to the difference between the quantization axes. In the Bloch sphere, this can be represented by rotations of the state vector around the second quantization axis. After half a period (orange arrow), the state projection on the quantization axis of the initial quantum dot differs maximally from that of the initial state. This sets the visibility M of the oscillations induced by the change of quantization axis.

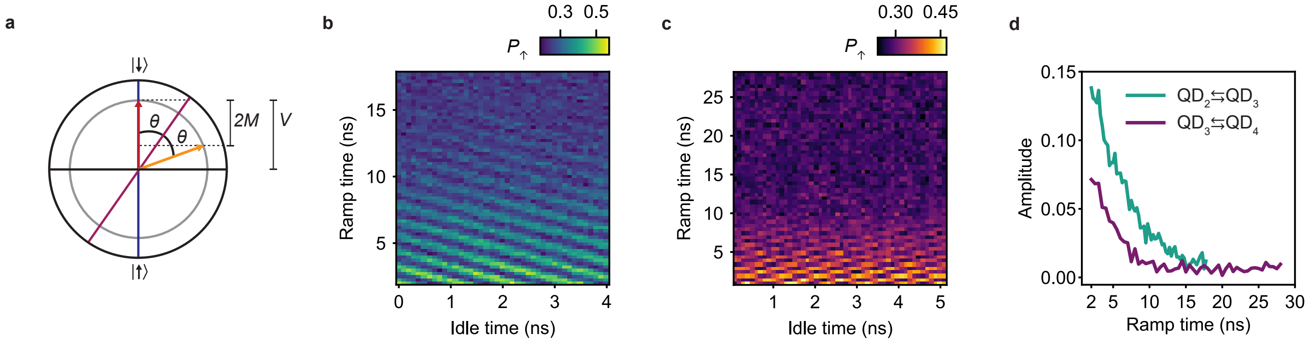

Supplementary Figure 4. **Estimation of the tilt angle based on the amplitude of the oscillations induced by the difference in quantization axis. a,** Geometric construction in the Bloch sphere allowing to determine the tilt angle θ between the quantization axes of adjacent quantum dots (blue and dark pink). θ is determined from the visibility M of the oscillations induced by the change in quantization axes and the visibility of the Rabi oscillations V. **b**, **c**, Oscillations induced while shuttling a qubit in a $|\downarrow\rangle$ state between QD_2 and QD_3 (b) and between QD_3 and QD_4 (c) for increasing ramp times. **d**, Amplitude of the oscillations as function of the ramp times.

In practice, this visibility is reduced due to imperfect initialization and readout. This can be taken into account by assuming that the state vectors have a norm $V < 1$ with V being the visibility of Rabi oscillations measured in the quantum dot where the shuttling experiment starts. We neglect relaxation which is irrelevant at the time scale of few nanoseconds [7] and thus assume that the norm of the vector state stays constant during the rotations. We find that:

$$\theta = \frac{1}{2}\arccos(1 - 2M/V) \text{ with } 0 \leq \theta \leq \pi. \tag{1}$$

We use this expression to evaluate θ_{23} (θ_{34}), the tilt angle between the quantization axes of QD_2 and QD_3 (QD_3 and QD_4). Supplementary Figure 4.b and c show the oscillations induced by the change in quantization axis as function of the pulse ramp time t_{ramp}. The amplitude $M/2$ of these oscillations is fitted and plotted in Supplementary Figure 4.d. As discussed in the main text, the amplitude of the oscillations drops rapidly to zero as t_{ramp} increases, because the shuttling becomes more adiabatic with respect to the difference in quantization axis. For the evaluation of θ we use the amplitude $M/2 = 0.14$ (0.07) of the oscillations at the shortest $t_{\mathrm{ramp}} = 2$ ns. We remark that there is no clear saturation of M at the smallest ramp times, which suggests that the shuttling process is still not fully diabatic and that higher visibilities could be achieved by shuttling faster. The Rabi oscillations for the driving of the qubit in QD_2 (QD_3) have a visibility of $V = 0.61$ (0.48) giving us $\theta_{23} \geq 42°$ ($\theta_{34} \geq 33°$). These large values for θ illustrate the strong influence of the local electric field on the direction of the quantization axis in germanium hole spin qubits operated with an in-plane external magnetic field.

B. Estimations based on fits with a four-level model

To get additional independent evaluations of the tilt angles, we can fit the evolution of the qubit resonance with a four-level model. To derive such a model, we consider a single hole in a germanium double quantum dot placed in an external magnetic field B. We assume that there is a finite tunnel coupling t_c between the two quantum dots QD_A and QD_B and their quantization axes are tilted with respect to each other by an angle θ. This last assumption is sufficient to take into account all effects of the spin-orbit interaction, providing a suitable basis transformation and a renormalization of the tunneling terms.

The system can be described in the basis $\{|A,\uparrow_A\rangle, |A,\downarrow_A\rangle, |B,\uparrow_A\rangle, |B,\downarrow_A\rangle\}$, where 'A' or 'B' indicates the position of the hole (in quantum dot QD_A or QD_B) and $\uparrow_A$ or $\downarrow_A$ specifies its spin states in the frame of quantum dot A. Its Hamiltonian is then given by:

$$H_{\mathrm{model}} = H_{\mathrm{charge}} + H_{\mathrm{Zeeman}} = \begin{pmatrix} \epsilon & 0 & t_c & 0 \\ 0 & \epsilon & 0 & t_c \\ t_c & 0 & -\epsilon & 0 \\ 0 & t_c & 0 & -\epsilon \end{pmatrix} + \frac{1}{2}B\mu_B \begin{pmatrix} g_A(\epsilon) & 0 & 0 & 0 \\ 0 & -g_A(\epsilon) & 0 & 0 \\ 0 & 0 & g_B(\epsilon)\cos(\theta) & g_B(\epsilon)\sin(\theta)e^{i\varphi} \\ 0 & 0 & g_B(\epsilon)\sin(\theta)e^{-i\varphi} & -g_B(\epsilon)\cos(\theta) \end{pmatrix}, \quad (2)$$

where ϵ is the detuning energy of the double quantum dot system (taken as zero at the charge transition), μ_B is the Bohr magneton and g_i are the g-factors in the different quantum dots, φ is the azimuthal angle between the two quantization axes. Note that, with this convention, ϵ corresponds to half of the difference between the electrochemical potentials of the two quantum dots and there is a tunnel gap of $2t_c$ at the anticrossing. We remark that this model is similar to that of a flopping-mode qubit [1]. Diagonalizing the Hamiltonian, we obtain the qubit resonance frequency f_L given by:

$$f_L = \frac{\mu_B B}{h} \frac{\sqrt{(2\epsilon^2 + t_c^2)(g_A(\epsilon)^2 + g_B(\epsilon)^2) + 2\epsilon(g_B(\epsilon)^2 - g_A(\epsilon)^2)\sqrt{\epsilon^2 + t_c^2} + 2g_A(\epsilon)g_B(\epsilon)t_c^2\cos(\theta)}}{2\sqrt{\epsilon^2 + t_c^2}}, \quad (3)$$

The evolution of f_L along the detuning axes can then be fitted to extract the tilt angles and the tunnel couplings between neighbouring quantum dots. For this purpose, we first express the detuning energies in terms of gate voltages as $\epsilon_{23} = -\frac{\eta_{23}}{2}(vP_3 - vP_3^0)$ and $\epsilon_{34} = -\frac{\eta_{34}}{2}(vP_4 - vP_4^0)$ where $\eta_{23} = 0.166$ meV/mV and $\eta_{34} = 0.150$ meV/mV are the effective lever arms along the detuning axis. They are defined as $\eta_{23} = \beta_3 + \beta_2\gamma_{23}$ and $\eta_{34} = \beta_4^* + \beta_3^*\gamma_{34}$ where $\beta_2 = 0.084$ meV/mV, $\beta_3 = 0.080$ meV/mV ($\beta_3^{(*)} = 0.084$ meV/mV, $\beta_4^{(*)} = 0.075$ meV/mV) are the virtual gate lever arms measured nearby the QD_2-QD_3 (QD_3-QD_4) charge transition via photon-assisted tunnelling experiments [8] and where $\gamma_{23} = |\Delta vP_2/\Delta vP_3|$=1.026 ($\gamma_{34} = |\Delta vP_3/\Delta vP_4| = 0.889$) are the slopes of the detuning axis. We extract the evolution of f_L as function of vP_3 (vP_4) from the data displayed in Supplementary Figure 5.a-b (Supplementary Figure 6.a-c) and fit it with eq.(3).

Supplementary Figure 5.c-d display the evolution of f_L along the ϵ_{23} detuning axis which is fitted to the above model assuming a linear dependence of g with vP_3. We observe that the model reproduces well the measured evolution. This allows to estimate an interdot tunnel coupling t_c of 4.4 ± 0.2 GHz and a tilt angle θ_{23} of $51.8 \pm 0.7°$. The uncertainties correspond to one standard deviation from the best fit. This evaluation is consistent with the lower bound found using the previous method.

Supplementary Figure 6.d-e display the evolution of f_L along the ϵ_{34} detuning axis. In this case, fitting the data does not allow to extract the tilt angle, even if we assume a quadratic dependence of the g-factor with the gate

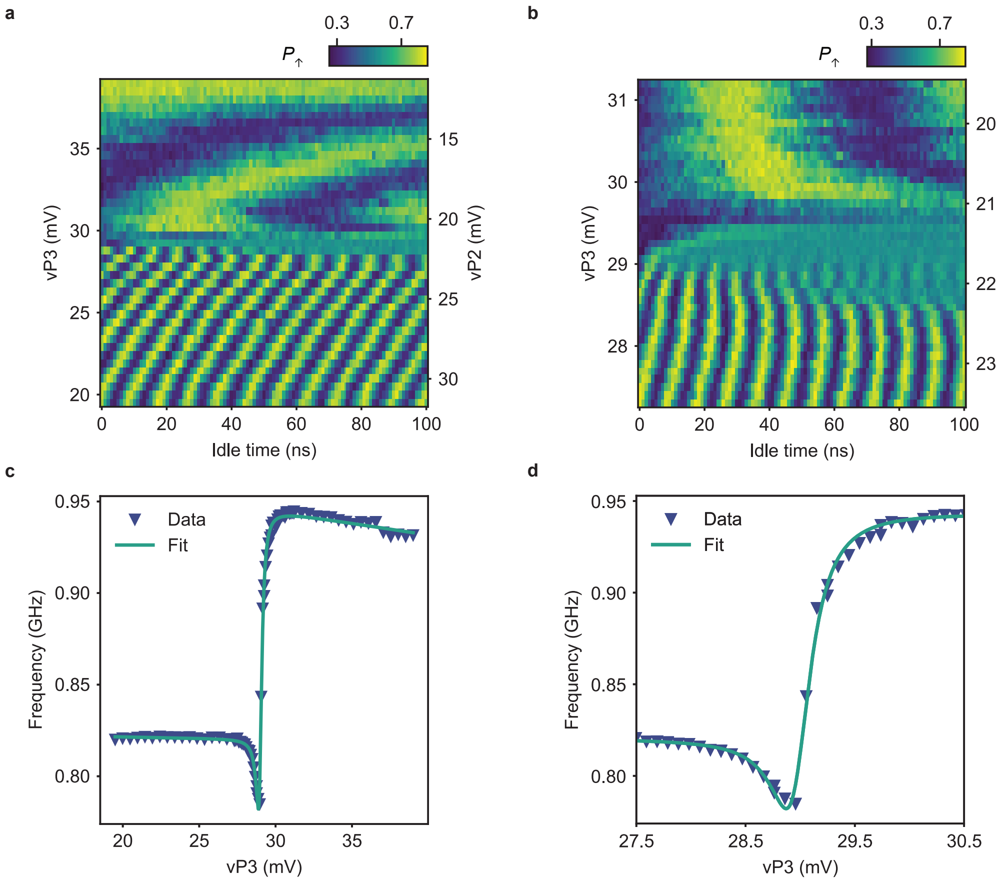

Supplementary Figure 5. **Evaluation of the tilt angle between QD$_2$ and QD$_3$ quantization axes using a four-level model.** **a**, Free evolution experiments for shuttling a qubit in superposition state between QD$_2$ and QD$_3$ back-and-forth. The superposition state is prepared in QD$_2$. **b**, Zoom-in on the vicinity of the charge transition. The two data sets are identical to those displayed in Fig. 1.h. **c**, **d**, Resonance frequency extracted from the oscillations along the detuning axis in (a) and (b) and fit with the model of eq. (3).

voltage. Indeed, for $0° \leq \theta \lesssim 40°$, the shape of f_L curve is nearly solely determined by the tunnel coupling and the variation of the g-factor with vP$_4$. Consequently, the data can be equally well fitted by models where θ_{34} is fixed at 0°, 10°, 20°, 30°or 40°. This leads to such a large uncertainty on the value of θ_{34} that it prevents us from extracting it. Nevertheless, the tunnel coupling between QD$_3$ and QD$_4$ can still be estimated from these fits and, for θ_{34} fixed to 40°(30°), we find $t_\mathrm{c} = 8 \pm 1$ ($t_\mathrm{c} = 6.2 \pm 0.8$) GHz.

What does become clear, however, is that we cannot obtain proper fits of the data with model where θ_{34} is fixed to values larger than 40°. The underlying reason appears when plotting the expected evolution of f_L in such model: for $\theta_{34} \gtrsim 50°$, f_L should display a minimum that we do not observe experimentally. This suggests that θ_{34} is lower than 50°.

This analysis also allows us to estimate the degree of adiabaticity of the charge transfers between the neighbouring quantum dots i and j. For that, we use the Landau-Zener formula $P_\mathrm{LZ}^{ij} = \exp(\frac{-2\pi t_\mathrm{c}^2 t_\mathrm{ramp}}{2\hbar|\Delta\epsilon_{ij}|})$ that gives us the probability of having a transition to the excited charge state while changing the detuning linearly by $|\Delta\epsilon_{ij}| = \frac{\eta_{ij}}{2}|\Delta\mathrm{vP}_{ij}|$ in a time t_ramp. We emphasize that the factor 2 in front of $\Delta\epsilon_{ij}$ comes from our definition of ϵ_{ij}. Taking $\Delta\mathrm{vP}_3 = 19.5$ mV for shuttling between QD$_2$ and QD$_3$, $\Delta\mathrm{vP}_4 = 18$ mV for shuttling between QD$_3$ and QD$_4$ and $t_\mathrm{ramp} = 4$ ns, we find $P_\mathrm{LZ}^{23} \simeq 2 \times 10^{-2}$ and $P_\mathrm{LZ}^{34} \simeq 2 \times 10^{-7} (9 \times 10^{-5})$. The values obtained for the shuttling between QD$_3$ and QD$_4$ suggest that the charge transfer between these two quantum dots is adiabatic. In contrast, there is non-negligible probability of exciting higher charge states while shuttling between QD$_2$ and QD$_3$ with these settings.

To improve the fidelity of the shuttling process between QD$_2$ and QD$_3$, we increased the tunnel coupling by lowering the barrier gate voltage vB$_{23}$ from -40 mV to -75 mV. Supplementary Figure 7 shows the results of similar analysis performed after lowering the barrier gate voltage. Fitting the evolution of the resonance frequency along the detuning

axis, we find that $t_c = 16.1 \pm 0.6$ GHz and $\theta_{23} = 54.2 \pm 0.6°$. For these experiments, $\eta_{23} = 0.164$ and $\Delta vP_3 = 24$ mV, thus we find $P_{LZ}^{23} \simeq 2 \times 10^{-19}$. In this gate voltage configuration, the shuttling process is fully adiabatic with respect to the charge degree of freedom. Consequently, we used these barrier gate voltage settings to have better shuttling performance and, in particular, for the experiments presented in Fig. 3 and Fig. 4 of the main text.

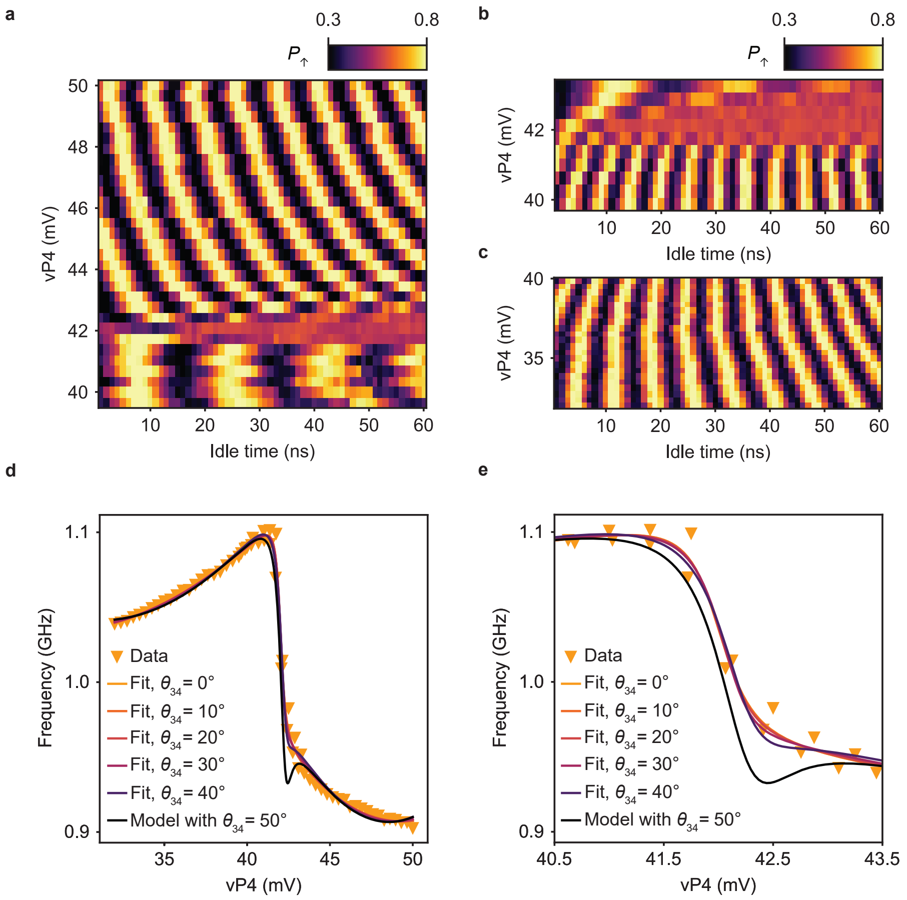

Supplementary Figure 6. **Evaluation of the tilt angle between QD_3 and QD_4 quantization axes using a four-level model.** **a**, **b**, **c**, Free evolution experiments for the adiabatic shuttling of a qubit in a superposition state between QD_3 and QD_4 back-and-forth. In (a) the qubit is prepared in superposition in QD_4, while in (b) and (c) the superposition state is prepared in QD_3. **d**, **e**, Evolution of the resonance frequency along the detuning axis, extracted from the oscillations in (a), (b) and (c), and fits with models of eq. (3) where the tilt angle is fixed. The expected evolution for $\theta_{34} = 50°$ is computed using the parameters extracted from the fit with $\theta_{34} = 40°$.

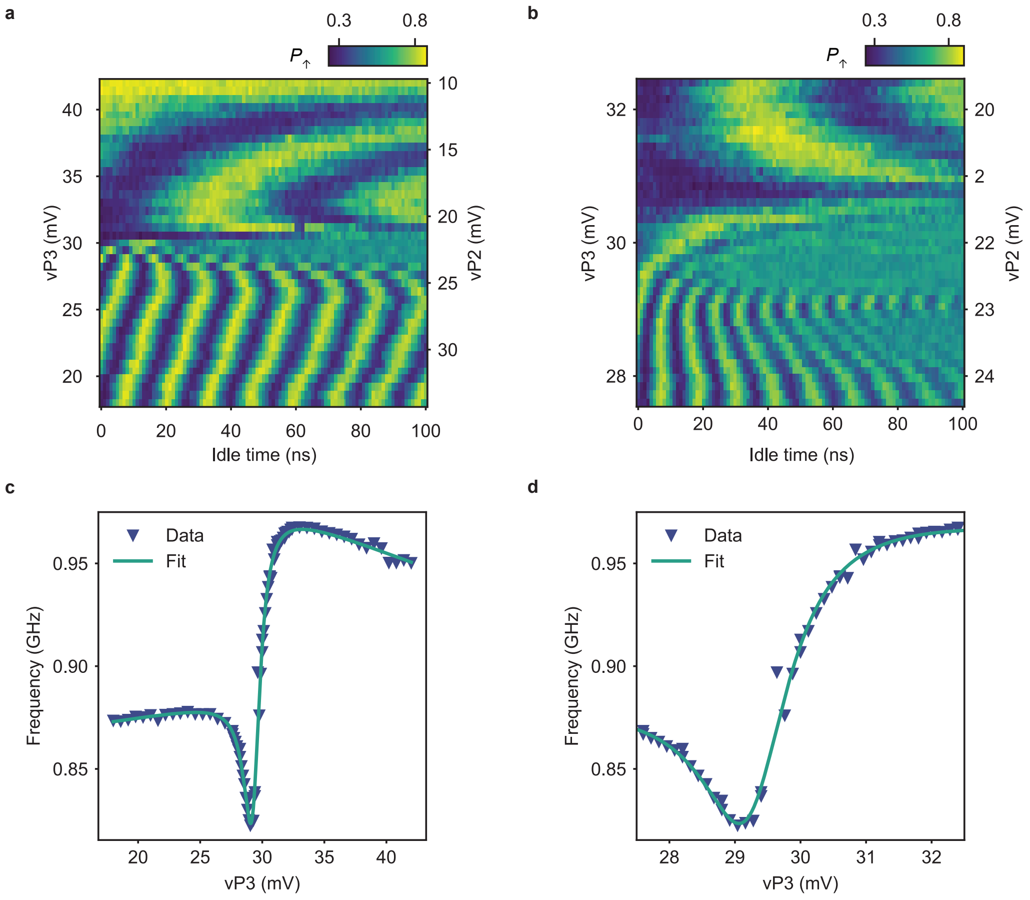

Supplementary Figure 7. **Evaluation of the tilt angle between QD_2 and QD_3 quantization axes using a four-level model at a lower barrier gate voltage.** **a**, Free evolution experiments for shuttling a qubit in a superposition state between QD_2 and QD_3 back-and-forth. The superposition state is prepared in QD_2. **b**, Zoom-in on the vicinity of the charge transition. **c**, **d**, Resonance frequency extracted from the oscillations along the detuning axis extracted from (a) and (b) and fit with the model of eq. (3). Compared to Supplementary Figure 5, here the barrier gate voltage vB_{23} is lower (-75 mV instead of -40 mV) leading to a higher tunnel coupling. These settings correspond to the settings used to acquire the data displayed in Fig. 3 and Fig. 4 of the main text.

Supplementary Note 3. Shuttling as a function of time and dephasing times

To get some insights on how the shuttling performance compare to the typical coherence times in the system, we plot in Supplementary Figure 8 the qubit dephasing times T_2^* alongside the results of the shuttling experiments as function of time. We evaluate the T_2^* of a static qubit at the locations in the charge stability diagrams corresponding to the starting and the end points of the shuttling pulses. The T_2^* values are measured using a standard Ramsey protocol. The resulting oscillations are fitted by $A\cos(2\pi t f + \varphi_0)\exp(-(t/T_2^*)^\alpha) + A_0$ allowing to extract both T_2^* and the decay coefficients α. The corresponding data and fits are shown in Supplementary Figure 8.a-b.

Moreover, for all the shuttling processes, we calculate for each number of shuttling events n the total time between the two $\pi/2$ pulses of the Ramsey shuttling experiments. Supplementary Figure 8.c-f show the results of shuttling experiments used to quantify the performances. These data are identical to those shown in Fig. 3. and 4. of the main text, but the amplitude decay is shown as a function of the time duration of the shuttling experiments.

An overview of the fit parameters is shown in Supplementary Table 2. Since the total measurement time for the shuttling experiments (several thousands of seconds) is very different from that for the Ramsey experiments (several hundreds of seconds), and therefore the type and amount of noise integrated are different, some caution is required when comparing the decay parameters. However, it is clear that the dephasing times of static and moving qubits are of the same order of magnitude.

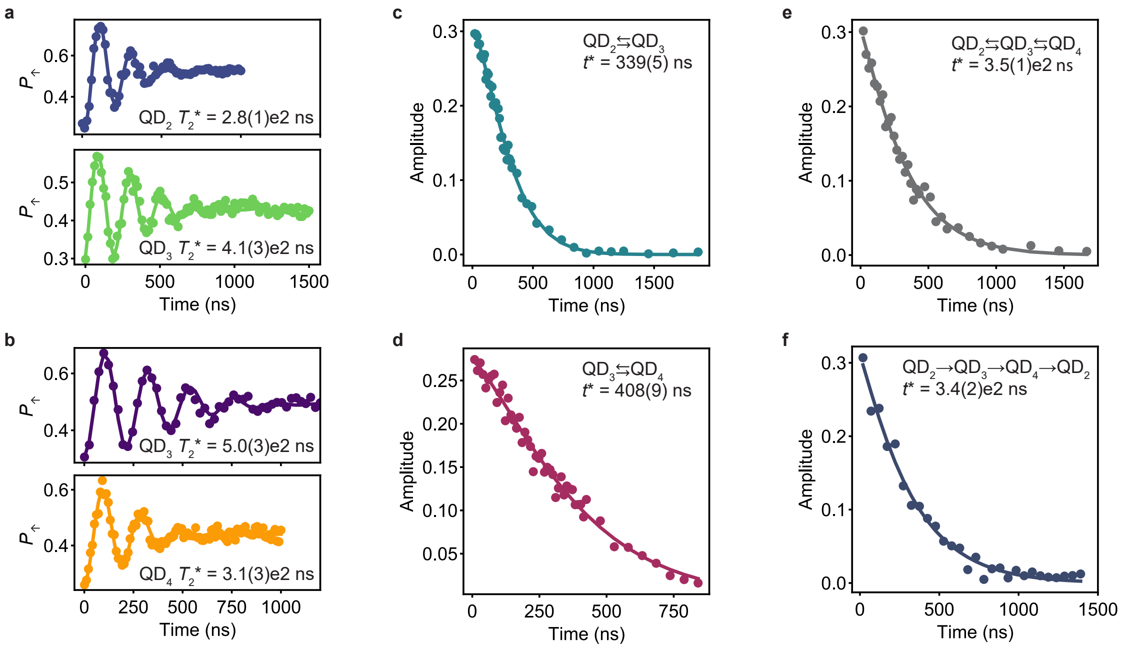

Supplementary Figure 8. **Comparison of the dephasing times for static and shuttled qubits.** **a**, **b**, Results of Ramsey experiments for a spin in QD_2 and QD_3 (a) (QD_3 and QD_4 in (b)) at the same plunger and barrier gate voltages settings as the shuttling experiments between QD_2 and QD_3 (a) (QD_3 and QD_4 in (b)). **c**, **d**, The result of the shuttling experiment, to assess the shuttling performance with a superposition state (without echo pulse), between QD_2 and QD_3 (c) (QD_3 and QD_4 in (d)), same as in Fig. 3g (3h) of the main text. However, here the amplitude is plotted as a function of the total time between the two $\pi/2$-pulses. **e**, **f**, Similar to (a) and (b), but for the corner shuttling and the triangular shuttling. The gate voltage settings for these experiments are the same as for the Ramsey experiments shown in (a) for QD_2 and QD_3, the T_2^* of QD_4 at the relevant gate voltage settings can be found in Supplementary Table 2. The uncertainties indicate one standard deviation from the best fits.

Supplementary Note 4. Adiabatic shuttling

For completeness, we also investigate the performance of the shuttling processes when the shuttling pulses are adiabatic, i.e. when there is no spin rotation induced by the difference between the quantization axes of the quantum

Shuttling process	T_2^* (ns) in QD_2	α in QD_2	T_2^* (ns) in QD_3	α in QD_3	T_2^* (ns) in QD_4	α in QD_4	t^* (ns) for shuttling	α for shuttling
$QD_2 \rightleftarrows QD_3$	$(2.8\pm0.1)\times10^2$	1.8 ± 0.2	$(4.1\pm0.3)\times10^2$	1.6 ± 0.2	N. A.	N. A.	339 ± 5	1.41 ± 0.05
$QD_3 \rightleftarrows QD_4$	N. A.	N. A.	$(5.0\pm0.3)\times10^2$	1.8 ± 0.3	$(3.1\pm0.2)\times10^2$	1.8 ± 0.2	408 ± 9	1.30 ± 0.07
Corner shuttling	$(2.8\pm0.1)\times10^2$	1.8 ± 0.2	$(4.1\pm0.3)\times10^2$	1.6 ± 0.2	$(3.1\pm0.3)\times10^2$	1.4 ± 0.3	$(3.5\pm0.1)\times10^2$	1.13 ± 0.07
Triangular shuttling	$(2.8\pm0.1)\times10^2$	1.8 ± 0.2	$(4.1\pm0.3)\times10^2$	1.6 ± 0.2	$(3.1\pm0.3)\times10^2$	1.4 ± 0.3	$(3.4\pm0.2)\times10^2$	1.11 ± 0.08

Supplementary Table 2. **Dephasing times and decay coefficients for static and shuttled qubits.** The dephasing times T_2^* for static qubits are measured with standard Ramsey experiments (data shown Supplementary Figure 8), performed at the starting and the end points of the shuttling pulses. The dephasing time t^* for shuttled qubits are extracted by fitting the amplitude as a function of the total time, as shown in Supplementary Figure 8. The uncertainties indicate one standard deviation from the best fits. The voltages applied on the barrier gates vary between experiments, which can lead to different T_2^* and α values for a static qubit in a given quantum dot.

dots. Supplementary Figure 9 shows the results of such investigations for the shuttling of basis states and for the shuttling of superposition states. In both cases, we obtain significantly lower performance compared to those achieved with diabatic pulses (see Figure 3 in the main text). According to our findings, dephasing can largely explain this difference in performance for the coherent shuttling experiments. As the time required for each shuttling event is increased in the adiabatic experiments, the qubit experiences more dephasing during each shuttling step and the phase coherence is lost after a smaller number of shuttling steps n. The use of echoing pulses allows us to get an improvement of the coherent shuttling performance by a factor 6 to 8, larger than those obtained for diabatic shuttling.

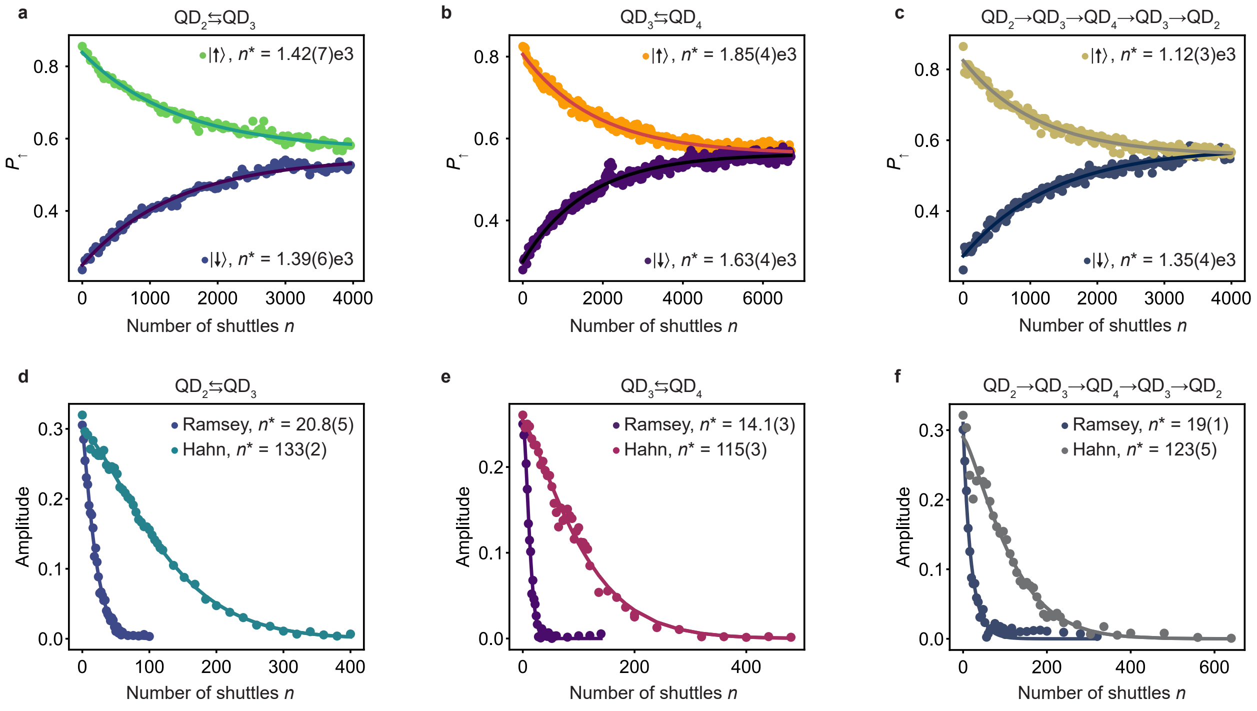

Supplementary Figure 9. **Performance of adiabatic shuttling.** **a**, **b**, **c**, Spin polarization as a function of the number of shuttling steps n for a qubit initialized in the basis states. **d**, **e**, **f**, Amplitude as a function of the number of shuttling steps n for qubits initialized in a superposition state, without (Ramsey) and with an echo pulse (Hahn).

For shuttling basis states, the lower performance suggests that the probability of having a spin-flip during a shuttle increases if the latter is performed adiabatically. This could originate from the longer time spent in the vicinity of the charge transition, where spin randomization induced by charge noise is enhanced [6]. Overall, the data in Supplementary Figure 9 clearly show that an approach based on diabatic spin shuttling is preferable for hole spin qubits in germanium.

Supplementary Note 5. Charge stability diagram of pair QD_2-QD_4 and triangular shuttling

The charge stability diagram of the quantum dot pair QD_2-QD_4, measured in a configuration identical to that of the triangular shuttling, is displayed in Supplementary Figure 10. No clear interdot charge anticrossing is visible, which suggests that the tunnel coupling between the two quantum dots is very low. This is expected, considering the device geometry, and it forces us to split the final pulse for the triangular shuttling in two parts. As depicted in Supplementary Figure 10, the voltages are first changed to bring the system close to the (1100)-(1001) degeneracy point before applying a second pulse that brings the system in the (1100) charge state. This reduces the probability that we excite the (1101) charge state, while transferring the qubit.

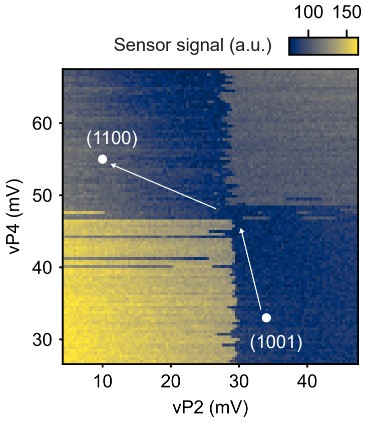

Supplementary Figure 10. **Charge stability diagram of quantum dot pair QD_2-QD_4.** No clear interdot transition can be distinguished. The shuttling of a spin qubit from QD_2 to QD_4 is performed using two voltage pulses (white arrows). The labels $(N_1N_2N_3N_4)$ represent the charge occupation in the quantum dots.

Supplementary Note 6. Optimization of the shuttling pulses to mitigate the effects of the spin-orbit interaction

In this section, we illustrate and discuss the importance of careful pulse optimization. Supplementary Figure 11 shows the results of experiments where we probe the performance of the coherent shuttling between QD_2 and QD_3 using the Ramsey sequence depicted in Fig. 3.a. The detuning pulses used for all these experiments are identical, except for the idle time t_{idle} in QD_3 (idle time 2 in Fig. 3.b). This idle time in QD_3 was optimized to 0.95 ns for the experiments displayed in the main text.

We observe that the evolution of amplitudes extracted at the end of the shuttling sequence is strongly dependent on the idle time in QD_3. For $t_{\mathrm{idle}} = 0.9$ and $t_{\mathrm{idle}} = 1$ ns, which are close to the optimum, the amplitude shows a smooth and progressive decay. When t_{idle} is increased, oscillations of the amplitude as function of the number of shuttling steps n appear and their periodicity varies with t_{idle}. These oscillations witness the rotations induced by the change of quantization axes, which are imperfectly compensated for $t_{\mathrm{idle}} \geq 1.1$ ns. They lead to coherent errors after each shuttling event, which add up, and significantly modify the state of the qubit. For example, for $t_{\mathrm{idle}} = 1.6$ ns, the superposition state is virtually transformed to a spin basis state after a few shuttling rounds. This emphasizes the necessity of optimizing the voltage pulses to compensate for the effect of rotations induced by the spin-orbit interaction.

The optimized idle times for each shuttling process can be found by performing measurements similar to those displayed in Supplementary Figure 11, and by looking for a regular decay of the amplitude as function of n. This optimization can also be done by studying the decay of the spin-up probabilities in spin basis state shuttling experiments.

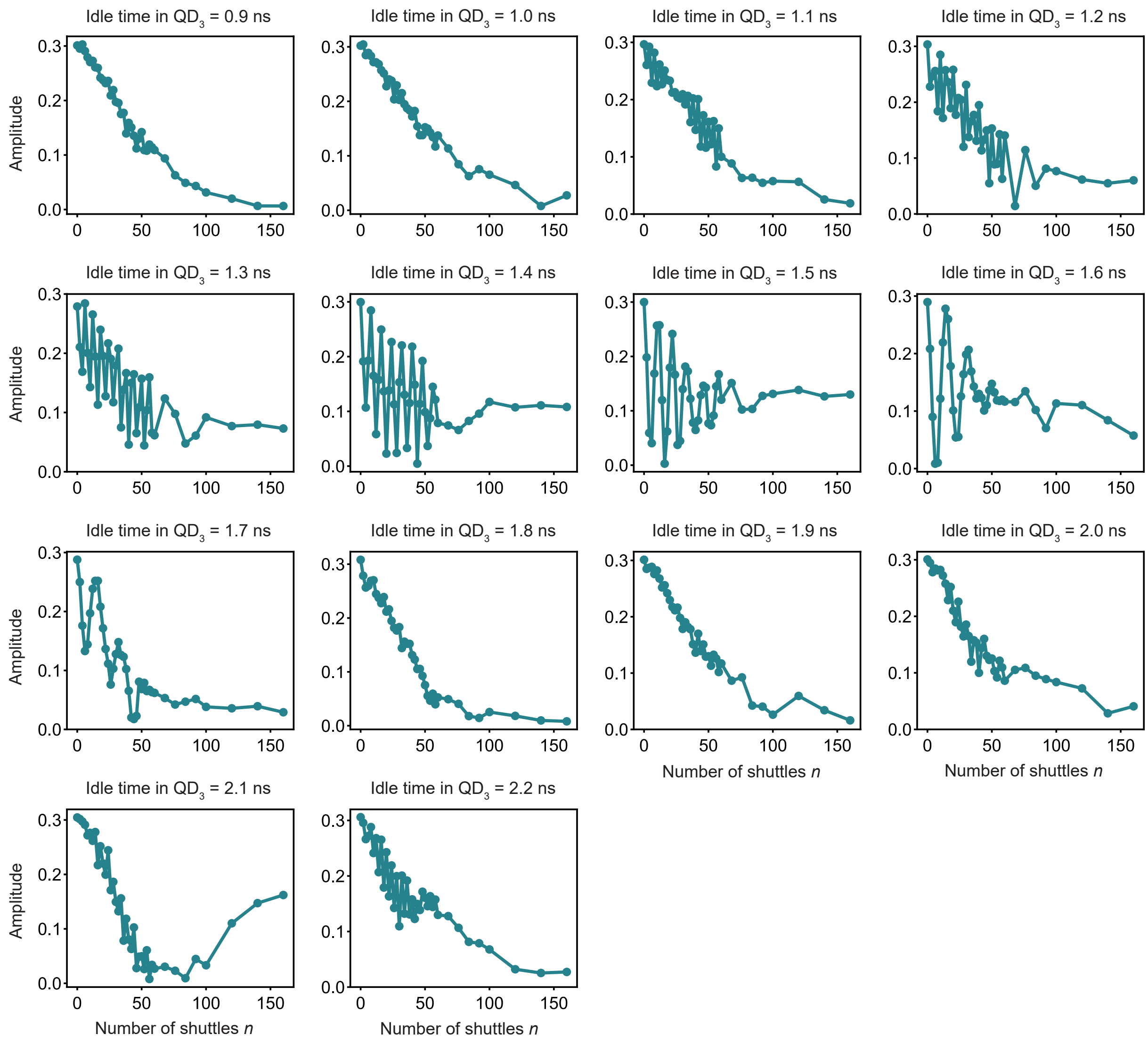

Supplementary Figure 11. **Signatures of non-optimized idle times in Ramsey shuttling experiments.** Results of coherent shuttling experiments between QD_2 and QD_3 obtained using Ramsey sequences. The idle time spent in QD_3 is different for the results shown in the different subplots, as indicated by the titles. For non-optimized idle times, oscillations of the amplitude as function of the number of shuttles n appear and the amplitude can saturate to a non-zero value at large n.

Supplementary Note 7. Qubit dynamics during coherent shuttling experiments for non-optimized idle times

In Supplementary Figure 11, we see that for non-optimized idle times, like $t_{\text{idle}} = 1.5$ ns, the amplitude can saturate to a finite value at large n. This is in contrast to what we observe for optimized idle times $t_{\text{idle}} = 0.9$ and $t_{\text{idle}} = 1$ ns, which decay to zero. To understand this feature, we carry out simulations of the dynamics of a qubit initialized in the $\frac{|\downarrow\rangle - i|\uparrow\rangle}{\sqrt{2}}$ superposition state which is shuttled between two neighboring quantum dots. Each shuttling step is modelled by a rotation. This rotation arises from the precession around the quantization axis of the quantum dot towards which the qubit is shuttled. We also calculate for every even n the expected measurement result, i.e. the amplitude of the $P_{\uparrow}$ oscillations that appear when the phase ϕ of the second $\pi/2$ pulse is varied. This is shown in Supplementary Figure 12.c, with two examples corresponding to a non-optimized idle time and an optimized idle time.

Supplementary Figure 12.a displays the trajectory in the Bloch sphere of the qubit for the first 14 shuttling steps, in the reference frame of the quantum dot where the shuttling experiment starts. The different states of the qubit map a

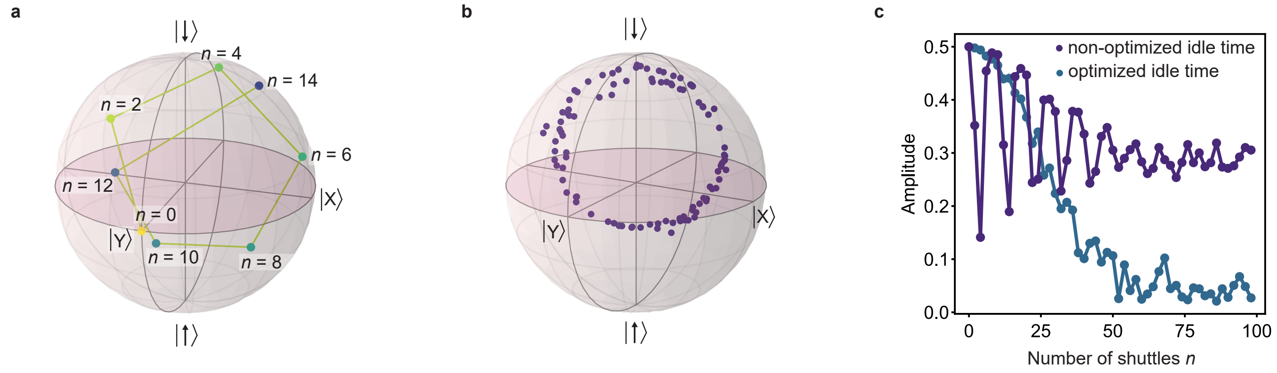

Supplementary Figure 12. **Simulation of the effect of non-optimized idle times.** **a**, Distribution of the qubit states on the Bloch sphere after an even number of shuttles, for an non-optimized idle time. **b**, Spread of the qubit state after a large number of shuttles, when the qubit is dephased. **c**, Simulated measurement results, i.e. amplitude of the oscillations appearing while varying the phase of the second $\pi/2$ pulse, as a function of n, for a non-optimized idle time and an optimized idle time.

circle which is tilted compared to the equator. The product of the two rotations generated by shuttling back-and-forth is equivalent to a single rotation around a fixed axis. Consequently, multiple shuttling cycles can be seen as successive rotations around this fixed axis which elucidates the trajectory observed in the Bloch sphere. This also explains the oscillations of the amplitude as function of n seen in Supplementary Figure 11, as the distance between origin and the projection of the state on xy-plane can vary significantly depending on the number of shuttles for an non-optimized idle time. In contrast, when the idle times are well-optimized, the qubit states are on the equator of the Bloch sphere and no oscillations of the amplitude with n can be observed.

Next, we include the effects of dephasing in the simulations, by assuming that the qubit frequencies fluctuate between repetitions of a given experiment with a fixed n. We observe that the state of the qubit is spread along a circle with a distribution that becomes more uniform as n increases, meaning when the qubit experiences more dephasing. An example is shown in Supplementary Figure 12.b for $n = 98$, corresponding to the data shown in Supplementary Figure 12.c. The center of the circle, which is equivalent to the statistical average of the qubit state when the qubit is completely dephased, is not on the equator on Bloch sphere. This explains the finite amplitude observed in the measurements at large n. Except for the revival of the amplitude observed for $t_{\mathrm{idle}} = 2.1$ ns, these simulations capture most of the features observed in Supplementary Figure 11.

Supplementary Note 8. Modelling of the qubit dynamics

A. Model used and underlying assumptions

In general, the quantum process of $2n$ shuttles, shuttling between QD_2 and QD_3 back-and-forth n times, is given by the sequential application of the individual processes:

$$\mathcal{U}_{2n \text{ shuttles}} = \langle \prod_{j=0}^{n} \mathcal{U}^{(j)}_{\mathrm{QD2}} \mathcal{U}^{(j)}_{\mathrm{r,QD2}} \mathcal{U}^{(j)}_{\mathrm{QD3}} \mathcal{U}^{(j)}_{\mathrm{r,QD3}} \rangle \,, \tag{4}$$

where $\mathcal{U}^{(j)}_{\mathrm{QD2(QD3)}}$ is the j-th superoperator describing the dynamics in quantum dot $\mathrm{QD}_2(\mathrm{QD}_3)$, $\mathcal{U}^{(j)}_{\mathrm{r,QD2(r,QD3)}}$ is the j-th superoperator describing the dynamics of ramping to quantum dot QD_2 (QD_3), and $\langle\cdot\rangle$ denotes averaging over different noise initializations. The dynamics within the qubit subspace without decoherence is given by:

$$U_{\mathrm{QD2}} = \exp\left(-i\frac{\phi_{\mathrm{QD2}}}{2}\sigma_z\right) \tag{5}$$

$$U_{\mathrm{r,QD2}} U_{\mathrm{QD3}} U_{\mathrm{r,QD3}} = \exp\left(-i\frac{\phi_{\mathrm{QD3}}}{2}(\cos(\theta_{23})\sigma_z + \sin(\theta_{23})\sigma_x)\right), \tag{6}$$

where θ_{23} is the effective tilt angle between the quantization axes that also takes the ramping time into consideration, $\phi_{\mathrm{QD2(QD3)}}$ are the effective phases accumulated around the corresponding quantization axis and $\sigma_{x,y,z}$ are the Pauli

matrices in QD_2. In the superoperator representation, the unitary dynamics can be conveniently expressed by:

$$\mathcal{U}_{\rm QD2} = U_{\rm QD2} \otimes U^*_{\rm QD2} \tag{7}$$
$$= e^{\mathcal{H}_{\rm QD2}}. \tag{8}$$

Here, $\mathcal{H}_{\rm QD2} = -i\phi_{\rm QD2}(\sigma_z \otimes \mathbf{1}_2 - \mathbf{1}_2 \otimes \sigma_z)/2$ and $\otimes$ denotes the Kronecker product. A similar expression holds for $\mathcal{U}_{\rm QD3}$. To describe the effect of decoherence we consider for each process low-frequency charge noise (modelled as quasistatic fluctuations of accumulated phases $\phi_{\rm QD2(QD3)} \to \phi_{\rm QD2(QD3)} + \delta\phi_{\rm QD2(QD3)}$), and high-frequency charge noise which causes dephasing and relaxation at the charge anticrossing. The latter noise sources are modelled within a standard Lindblad equation (allowing us to drop the index j) and described by the following Lindblad operators in superoperator representation using row-stacking convention [9]:

$$\mathcal{L}_{\rm QD2} = \begin{pmatrix} -\gamma^{\rm r}_{\rm QD2} & 0 & 0 & \gamma^{\rm r}_{\rm QD2} \\ 0 & -\gamma^{\rm r}_{\rm QD2} - \gamma^{\varphi}_{\rm QD2} & \gamma^{\rm r}_{\rm QD2} & 0 \\ 0 & \gamma^{\rm r}_{\rm QD2} & -\gamma^{\rm r}_{\rm QD2} - \gamma^{\varphi}_{\rm QD2} & 0 \\ \gamma^{\rm r}_{\rm QD2} & 0 & 0 & -\gamma^{\rm r}_{\rm QD2} \end{pmatrix}, \tag{9}$$

$$\mathcal{L}_{\rm r,QD2} = \begin{pmatrix} -\gamma^{\rm r}_{\rm r,QD2} & 0 & 0 & \gamma^{\rm r}_{\rm r,QD2} \\ 0 & -\gamma^{\rm r}_{\rm r,QD2} - \gamma^{\varphi}_{\rm r,QD2} & \gamma^{\rm r}_{\rm r,QD2} & 0 \\ 0 & \gamma^{\rm r}_{\rm r,QD2} & -\gamma^{\rm r}_{\rm r,QD2} - \gamma^{\varphi}_{\rm r,QD2} & 0 \\ \gamma^{\rm r}_{\rm r,QD2} & 0 & 0 & -\gamma^{\rm r}_{\rm r,QD2} \end{pmatrix}, \tag{10}$$

$$\mathcal{L}_{\rm QD3} = \left(R(\theta_{23}) \otimes R(-\theta_{23})^T\right) \begin{pmatrix} -\gamma^{\rm r}_{\rm QD3} & 0 & 0 & \gamma^{\rm r}_{\rm QD3} \\ 0 & -\gamma^{\rm r}_{\rm QD3} - \gamma^{\varphi}_{\rm QD3} & \gamma^{\rm r}_{\rm QD3} & 0 \\ 0 & \gamma^{\rm r}_{\rm QD3} & -\gamma^{\rm r}_{\rm QD3} - \gamma^{\varphi}_{\rm QD3} & 0 \\ \gamma^{\rm r}_{\rm QD3} & 0 & 0 & -\gamma^{\rm r}_{\rm QD3} \end{pmatrix} \left(R(-\theta_{23}) \otimes R(\theta_{23})^T\right), \tag{11}$$

$$\mathcal{L}_{\rm r,QD3} = \left(R(\theta_{23}) \otimes R(-\theta_{23})^T\right) \begin{pmatrix} -\gamma^{\rm r}_{\rm r,QD3} & 0 & 0 & \gamma^{\rm r}_{\rm r,QD3} \\ 0 & -\gamma^{\rm r}_{\rm r,QD3} - \gamma^{\varphi}_{\rm r,QD3} & \gamma^{\rm r}_{\rm r,QD3} & 0 \\ 0 & \gamma^{\rm r}_{\rm r,QD3} & -\gamma^{\rm r}_{\rm r,QD3} - \gamma^{\varphi}_{\rm r,QD3} & 0 \\ \gamma^{\rm r}_{\rm r,QD3} & 0 & 0 & -\gamma^{\rm r}_{\rm r,QD3} \end{pmatrix} \left(R(-\theta_{23}) \otimes R(\theta_{23})^T\right), \tag{12}$$

where $R(\theta) = e^{-i\theta\sigma_y/2}$ rotates the quantization axis by θ in the xz-plane. Here, $\gamma^{\varphi}_{\rm QD2(QD3)}$ is the dephasing rate and $\gamma^{\rm r}_{\rm QD2(QD3)}$ the relaxation/excitation rate for idling in $QD_2(QD_3)$ and $\gamma^{\varphi}_{\rm r,QD2(QD3)}$ is the dephasing rate and $\gamma^{\rm r}_{\rm r,\ QD2(QD3)}$ the relaxation/excitation rate for shuttling from QD_3 to QD_2 (QD_2 to QD_3). To simplify the expressions, we further assumed that the qubit is coupled to a hot qubit bath at the anticrossing [6] giving rise to equal relaxation and excitation rates. This assumption is justified by the fast randomization of the spin state at the anticrossing observed in Supplementary Figure 3.

The basic repetition, a 2-shuttle process, can now be described as a Markov chain:

$$\mathcal{U}_{2\text{ shuttles}} = \langle e^{\mathcal{H}_{\rm QD2}+\mathcal{L}_{\rm QD2}} e^{\mathcal{L}_{\rm r,QD2}} e^{\mathcal{H}_{\rm QD3}+\mathcal{L}_{\rm QD3}} e^{\mathcal{L}_{\rm r,QD3}} \rangle\,. \tag{13}$$

Here, we make the following assumptions. Firstly, high-frequency noise, causing relaxation and dephasing processes and described by $\gamma^{\varphi}_{\rm r,QD2(QD3)}$ and $\gamma^{,\rm r}_{\rm r,QD2(QD3)}$, is relevant only exactly at the anti-crossing and is instantaneous. Secondly, we neglect high frequency noise during the idling in QD_2 and QD_3 since idling dynamics is dominated by low-frequency noise. This allows us to add the coherent part of the dynamics during the ramp before (after) reaching the anti-crossing to the dynamics described by $\mathcal{H}_{\rm QD2}$ ($\mathcal{H}_{\rm QD3}$). We note that this is a good approximation if $\langle\phi_{\rm QD3}\rangle$ is close to an integer multiple of 2π, i.e. the shuttling rounds are well calibrated. Using the Zassenhaus expansion formula, we can now further approximate the 2-shuttle process by a product of two matrices:

$$\mathcal{U}_{2\text{ shuttles}} = \langle e^{\mathcal{C}} e^{\mathcal{D}} \rangle\,. \tag{14}$$

Here, the $\mathcal{C}$-matrix only consists of the average phase accumulated $\langle\phi_{\rm QD2}\rangle$ and describes a unitary process while all

decoherence is included in the D-matrix. For n being an even integer, a $2n$-shuttle process can then be written as:

$$\mathcal{U}_{2n\text{ shuttles}} = \left\langle \prod_{i=1}^{n/2} e^{\mathcal{D}} \begin{pmatrix} 1 & 0 & 0 & 0 \\ 0 & e^{-i\langle\phi_{\mathrm{QD2}}\rangle} & 0 & 0 \\ 0 & 0 & e^{i\langle\phi_{\mathrm{QD2}}\rangle} & 0 \\ 0 & 0 & 0 & 1 \end{pmatrix} e^{\mathcal{D}} \right\rangle, \tag{15}$$

with the decoherence matrix:

$$\mathcal{D} \approx \mathcal{L}_{\mathrm{r,QD2}} + \mathcal{L}_{\mathrm{r,QD3}} + \mathcal{H}_{\mathrm{QD2}} + \mathcal{H}_{\mathrm{QD3}} - \langle\mathcal{H}_{\mathrm{QD2}}\rangle\,. \tag{16}$$

B. Shuttling of spin basis states

The return probability of the basis states, which are identical for both basis states as the excitation and relaxation rate are assumed to be equal, can be computed from the superoperator $\langle\widetilde{\uparrow}|\mathcal{U}_{2n\text{ shuttle}}|\widetilde{\uparrow}\rangle$, where $|\widetilde{\uparrow}\rangle$ is the vectorized density matrix of the $|\uparrow\rangle$ basis state. We now consider two extreme cases, $\langle\phi_{\mathrm{QD2}}\rangle = m$ with even or odd m multiple of π. If m is an even multiple of π, we can simplify the expression to $\mathcal{U}_{2n\text{ shuttles}} = \langle e^{n\mathcal{D}}\rangle$, while for m being an odd multiple of π, the sequence corresponds to a Hahn echo experiment with a phase-flip operation instead of a bit-flip operation. In Supplementary Figure 13, we have simulated the basis state decay for varying m considering (a) uncorrelated and (b) correlated charge noise. While most cases shows only a single, and slow exponential decay, the special case of $m = 2\pi k$ (with k an integer) shows a fast initial Gaussian decay followed by a slow exponential decay. Regardless of the chosen value of m, the decay converges to $\langle\widetilde{\uparrow}|\mathcal{U}_{n\to\infty}|\widetilde{\uparrow}\rangle = \frac{1}{2}$. We find that for most m, the initial rapid decay is absent due to decoupling over potentially many shuttle rounds. Thus, the (partial) noise-decoupling effect occurs for most choices of the waiting times in the initial quantum dot. We find that the fast decay of the basis states can only be observed for $\langle\phi_{\mathrm{QD2}}\rangle = m \pm \delta$ with m being multiples of 2π and $\delta \leq 0.02\pi$. Since our experimental results do not show a fast Gaussian decay of the basis states, we believe that our timing is chosen such that the fast initial Gaussian dephasing is echoed out [10]. Consequently, we can fit the decay for the shuttling of spin basis states to an exponential decaying function $\exp(-n/n^*)$. We note that the final slow decay can either originate from spin randomization or from dephasing.

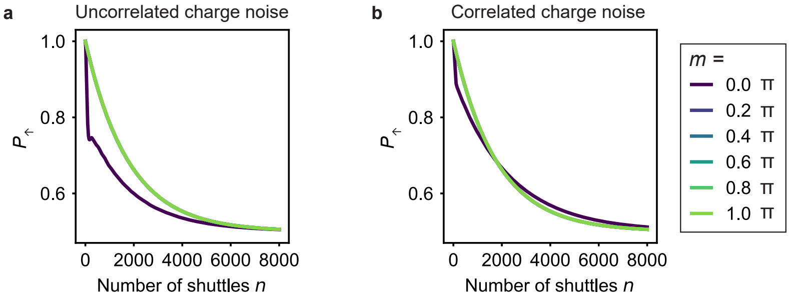

Supplementary Figure 13. **Simulation of the basis state decay.** Evolution of the spin-up probability $P_\uparrow$ as a function of the number of shuttling events simulated using Eq. (4) for **a**, uncorrelated and **b**, correlated quasistatic fluctuations for various $\langle\phi_{\mathrm{QD2}}\rangle = m$. For both correlated and uncorrelated noise, the fast initial decay vanishes, except when m is close to (a multiple of) 2π. In the simulation the following parameters were chosen: quantization axis difference $\theta_{23} = 52°$, high-frequency dephasing rate $\gamma^{\varphi}_{\mathrm{r,QD2}} = \gamma^{\varphi}_{\mathrm{r,QD3}} = 0.00125$, relaxation rate $\gamma^{\mathrm{r}}_{\mathrm{r,QD2}} = \gamma^{\mathrm{r}}_{\mathrm{r,QD3}} = 0.000125$, standard deviation of the quasistatic fluctuations $\sigma_{\phi_{\mathrm{QD2}}} = \sigma_{\phi_{\mathrm{QD3}}} = 0.004$. For uncorrelated fluctuations $\sigma_{\phi_{\mathrm{QD2}}} \to \sqrt{2}\sigma_{\phi_{\mathrm{QD2}}}$ to get identical free induction decays. These parameters lead to a pure dephasing after $n^* = 63$ shuttles in absence of decoupling.

C. Ramsey, Hahn-echo, and CPMG dynamical decoupling

The decay of the superposition states can be computed from the same superoperator via the amplitude

$$A = \frac{1}{2}\max_{\varphi}\left[\langle\widetilde{\varphi}_+|\mathcal{U}_{2n\text{ shuttle}}|\widetilde{\phi}_+\rangle - \langle\widetilde{\varphi}_-|\mathcal{U}_{2n\text{ shuttle}}|\widetilde{\phi}_+\rangle\right], \tag{17}$$

where $|\widetilde{\xi}_\pm\rangle$ is the vectorized density matrix of the state $|\xi_\pm\rangle = \frac{1}{2}(|\uparrow\rangle \pm e^{i\xi}|\downarrow\rangle)$ with $\xi = \varphi, \phi$. The initial phase of the superposition state is in general unknown because the clocks of the AWGs and the vector source (see section below) are not synchronized and thus the initial phase of the microwave source may vary for each single shot measurement of the experiment. However, the phase difference between initial and final state is fixed for each shot. We have numerically confirmed that the dynamics is only slightly affected by an additional averaging over the initial phase of the initial superposition state for $\langle\phi_{\text{QD2}}\rangle = m$ with m being close to multiples of 2π (same regime in which the fast initial decay can be observed for the basis state). For other choices, the initial phase has a negligible impact and is averaged out after a few shuttling rounds.

The quantum process of a dynamical decoupled $2n$ shuttles, shuttling between QD_2 and QD_3 back-and-forth n times, is given by the sequential application of the individual processes:

$$\mathcal{U}^{\text{Echo}}_{2n\text{ shuttles}} = \langle\prod_{j=0}^{n/2}\mathcal{U}^{(j)}_{\text{QD2}}\mathcal{U}^{(j)}_{\text{r,QD2}}\mathcal{U}^{(j)}_{\text{QD3}}\mathcal{U}^{(j)}_{\text{r,QD3}}\;\mathcal{U}_{\text{x},\pi}\prod_{j=n/2}^{n}\mathcal{U}^{(j)}_{\text{QD2}}\mathcal{U}^{(j)}_{\text{r,QD2}}\mathcal{U}^{(j)}_{\text{QD3}}\mathcal{U}^{(j)}_{\text{r,QD3}}\rangle, \tag{18}$$

$$\mathcal{U}^{\text{CPMG}}_{2n\text{ shuttles}} = \langle\prod_{j=0}^{n/4}\mathcal{U}^{(j)}_{\text{QD2}}\mathcal{U}^{(j)}_{\text{r,QD2}}\mathcal{U}^{(j)}_{\text{QD3}}\mathcal{U}^{(j)}_{\text{r,QD3}}\;\mathcal{U}_{\text{y},\pi}\prod_{j=n/4}^{3n/4}\mathcal{U}^{(j)}_{\text{QD2}}\mathcal{U}^{(j)}_{\text{r,QD2}}\mathcal{U}^{(j)}_{\text{QD3}}\mathcal{U}^{(j)}_{\text{r,QD3}}\;\mathcal{U}_{\text{y},\pi}\prod_{j=3n/4}^{n}\mathcal{U}^{(j)}_{\text{QD2}}\mathcal{U}^{(j)}_{\text{r,QD2}}\mathcal{U}^{(j)}_{\text{QD3}}\mathcal{U}^{(j)}_{\text{r,QD3}}\rangle, \tag{19}$$

where $\mathcal{U}_{\text{x,y},\pi} = \sigma_{x,y}\otimes\sigma^*_{x,y}$ are superoperator representation of the spin-flips around the x and y axis (here assumed to be error free). We note that here the CPMG sequence is simulated using $(\pi)_y$ decoupling pulses that are orthogonal to the initial state in the microwave frame. This corresponds to experiments under the assumption that the phase accumulated in the initial dot is not equal to $2k\pi$ (with k an integer). Only for the special case of a phase of $2k\pi$ (or close to $2k\pi$), we expect that the exact waiting times, the synchronization between the microwave source and the arbitrary waveform generators and the direction of the decoupling pulses will have an impact on the dynamics. For corner shuttling, shuttling between QD_2 to QD_3 to QD_4 and back n times, one needs to make the substitution:

$$\mathcal{U}^{(j)}_{\text{r,QD2}}\mathcal{U}^{(j)}_{\text{QD3}}\mathcal{U}^{(j)}_{\text{r,QD3}} \to \mathcal{U}^{(j)}_{\text{r,QD2}}\mathcal{U}^{(j)}_{\text{QD3}}\mathcal{U}^{(j)}_{\text{r,QD3}}\mathcal{U}^{(j)}_{\text{QD4}}\mathcal{U}^{(j)}_{\text{r,QD4}}\mathcal{U}^{(j)}_{\text{QD3}}\mathcal{U}^{(j)}_{\text{r,QD3}}, \tag{20}$$

where $\mathcal{U}^{(j)}_{\text{QD4}}$ is the j-th superoperator describing the dynamics in dot QD_4, $\mathcal{U}^{(j)}_{\text{r,QD4}}$ is the j-th superoperator describing the dynamics of ramping to quantum dot QD_4. The dynamics within the qubit subspace without decoherence is analogously given by:

$$\begin{aligned} U_{\text{r,QD2}}U_{\text{QD3}}U_{\text{r,QD3}}U_{\text{QD4}}U_{\text{r,QD4}}U_{\text{QD3}}U_{\text{r,QD3}} =& \exp\left(-i\frac{\phi_{\text{QD3,a}}}{2}(\cos(\theta_{23})\sigma_z + \sin(\theta_{23})\sigma_x)\right)\\ &\times\exp\left(-i\frac{\phi_{\text{QD4}}}{2}(\cos(\theta_{34}+\theta_{23})\sigma_z + \sin(\theta_{34}+\theta_{23})\sigma_x)\right)\\ &\times\exp\left(-i\frac{\phi_{\text{QD3,b}}}{2}(\cos(\theta_{23})\sigma_z + \sin(\theta_{23})\sigma_x)\right), \end{aligned} \tag{21}$$

where θ_{34} is the effective tilt angle between the quantization axes of QD_3 and QD_4 that also takes the ramping time into consideration, $\phi_{\text{QD3,a(b)}}$ is the effective phases accumulated in QD_3 before (after) shuttling to QD_4, and ϕ_{QD4} is the effective phases accumulated in QD_4.

Note that in general the quantization axes of QD_2, QD_3 and QD_4 do not need to lay in the same plane. To account for the misalignment we use the Euler-angle decomposition, i.e. by adding a rotation around the quantization axis of QD_3 before the shuttling to QD_4 and adding the inverse rotation after the shuttling back to QD_3. The additional phase commutes (by design) with the idling dynamics in QD_3. Consequently, the additional rotation can be added/subtracted from the rotation accumulated during idling in QD_3 (care has to be taken with the relaxation decay dynamics). Since we do not know the phase of the rotation in our experiment, we assume in our simulations that all three quantization axes are in the xz-plane. We have numerically confirmed that the dynamics is only mildly affected by this choice and only close to $\langle\phi_{\text{QD2}}\rangle = m$ with m being a multiple of 2π.

In Supplementary Figure 14, we have simulated for the corner shuttling the evolution of the coherence, i.e. the amplitude (see Eq. (17)) of the superposition state, for a (a) Ramsey, (b) Hahn-echo, and (c) CPMG dynamical decoupling sequence for various m using uncorrelated quasistatic charge noise. When m is a multiple of 2π, the dynamical decoupling for the CPMG sequence shows a significant improvement compared to the Hahn-echo. In contrast, when m is a multiple of π, a Hahn-echo sequence achieves a similar decoupling effect as the CPMG sequence because of the interplay between the phase and spin flips in QD_2. This could possibly explain the reduced effect of CPMG measured in corner shuttling. We note that simulations considering time-correlated phase fluctuations, instead of quasistatic phase fluctuations, show qualitatively similar results for the improvement of decoupling using CPMG sequences compared to Hahn-echo sequences.

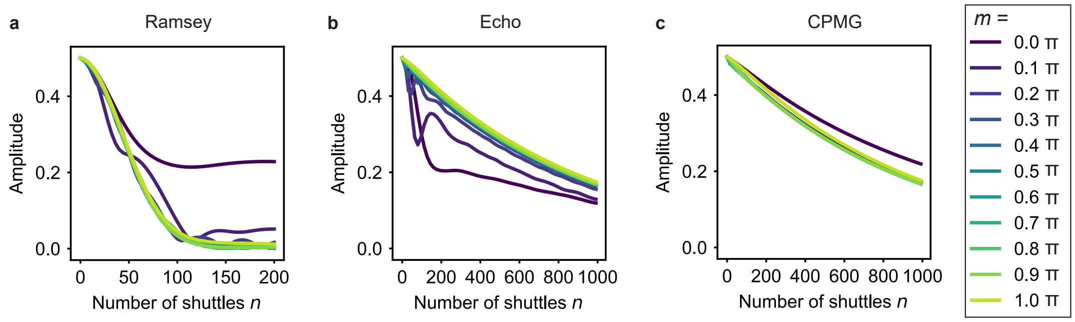

Supplementary Figure 14. **Simulation of the dynamics for the Ramsey, Hahn-echo and CPMG sequences for corner shuttling.** Evolution of the superposition state for corner shuttling as a function of the number of shuttling events using eq. (17) for uncorrelated quasistatic fluctuations for varying phases $\langle \phi_{\mathrm{QD2}} \rangle = m$ and assuming perfect single-qubit gates $\mathcal{U}_{x,y,\pi/2}$. The gain of implementing a CPMG dynamical decoupling sequence compared to an echo is negligible. In the simulation the following parameters were chosen: quantization axis differences $\theta_{23} = 52°$ and $\theta_{34} = 40°$, high-frequency dephasing rate $\gamma^{\varphi}_{\mathrm{QD2}} = \gamma^{\varphi}_{r,\mathrm{QD3}} = \gamma^{\varphi}_{r,\mathrm{QD4}} = 0.00125$, relaxation rate $\gamma^{\mathrm{r}}_{r,\mathrm{QD2}} = \gamma^{\mathrm{r}}_{r,\mathrm{QD3}} = \gamma^{\mathrm{r}}_{r,\mathrm{QD4}} = 0.000125$, standard deviation of the quasistatic fluctuations $\sigma_{\phi_{\mathrm{QD2}}} = \sigma_{\phi_{\mathrm{QD3}}} = \sigma_{\phi_{\mathrm{QD4}}} = 0.009$. These parameters lead to a pure dephasing after $n^* = 63$ shuttles in absence of decoupling. We note that the decay in these simulations are underestimated as they include only quasistatic noise.

Supplementary Note 9. Driving a spin qubit after shuttling to compensate unintended rotations

Since the rotations induced by diabatic shuttling are coherent, it should be possible to compensate these rotations by applying a microwave pulse. This requires the synchronisation of the internal clocks of the arbitrary waveform generators (AWGs) and the microwave source. Supplementary Figure 15.a shows the pulse sequence used to investigate this strategy. A spin prepared in the $|\downarrow\rangle$ state is shuttled back-and-forth once between QD_2 and QD_3. The idle time in QD_3 is purposely chosen such that the spin does not experience a 2π rotation around the tilted quantization axis. As a result, after the shuttling, the spin is not in the $|\downarrow\rangle$ state anymore. Back in QD_2, an EDSR pulse is applied of which the phase ϕ and duration t_{MW} are varied. Finally, the spin is readout.

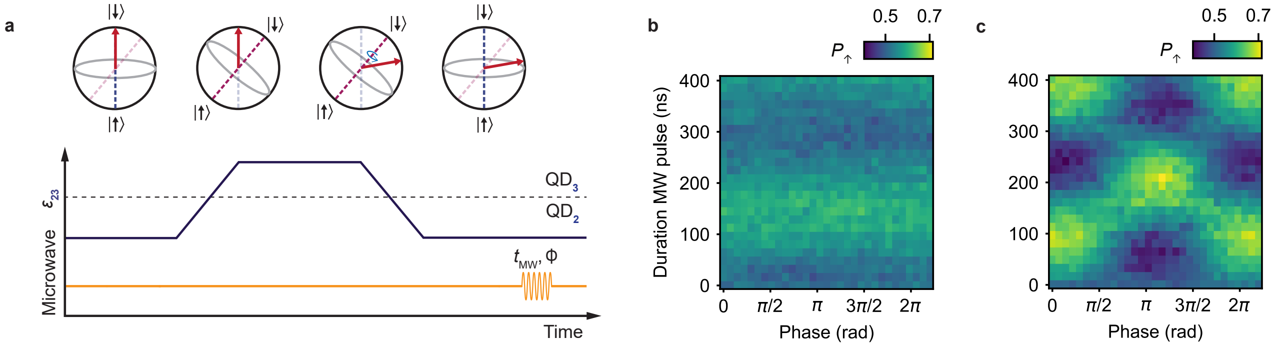

Supplementary Figure 15. **Compensating shuttling-induced oscillations with a final EDSR pulse.** **a**, Schematic of the pulse sequence used for testing the effect of an EDSR pulse after shuttling a spin qubit. A spin qubit is initialized in the $|\downarrow\rangle$ state and shuttled back-and-forth between QD_2 an QD_3. Finally, a microwave pulse of duration t_{MW} and phase ϕ is applied. **b**, Results of the measurement sequence depicted in (a), in the case where the clock of the AWGs and the microwave source are not synchronized. **c**, Results in the case where the clocks of the AWGs and the microwave source are synchronized. The reduced visibility of the oscillations in the spin-up probability, compared to the data presented in the main text, is due to a different tuning of the device.

[1] Benito, M., Croot, X., Adelsberger, C., Putz, S., Mi, X., Petta, J. R. & Burkard, G. Electric-field control and noise protection of the flopping-mode spin qubit. *Phys. Rev. B* **100**, 125430 (2019).
[2] Croot, X., Mi, X., Putz, S., Benito, M., Borjans, F., Burkard, G. & Petta, J. R. Flopping-mode electric dipole spin resonance. *Phys. Rev. Res.* **2**, 012006 (2020).
[3] Mutter, P. M. & Burkard, G. Natural heavy-hole flopping mode qubit in germanium. *Phys. Rev. Res.* **3**, 013194 (2021).
[4] Hu, R.-Z., Ma, R.-L., Ni, M., Zhou, Y., Chu, N., Liao, W.-Z., Kong, Z.-Z., Cao, G., Wang, G.-L., Li, H.-O. & Guo, G.-P. Flopping-mode spin qubit in a Si-MOS quantum dot. *Applied Physics Letters* **122**, 134002 (2023).
[5] Abadillo-Uriel, J. C., Rodríguez-Mena, E. A., Martinez, B. & Niquet, Y.-M. Hole spin driving by strain-induced spin-orbit interactions. *Phys. Rev. Lett.* **131**, 097002 (2023).
[6] Krzywda, J. A. & Cywiński, Ł. Interplay of charge noise and coupling to phonons in adiabatic electron transfer between quantum dots. *Phys. Rev. B* **104**, 075439 (2021).
[7] Lawrie, W. I. L., Hendrickx, N. W., van Riggelen, F., Russ, M., Petit, L., Sammak, A., Scappucci, G. & Veldhorst, M. Spin relaxation benchmarks and individual qubit addressability for holes in quantum dots. *Nano. Lett.* **20**, 7237–7242 (2020).
[8] Oosterkamp, T. H., Fujisawa, T., van der Wiel, W. G., Ishibashi, K., Hijman, R. V., Tarucha, S. & Kouwenhoven, L. P. Microwave spectroscopy of a quantum-dot molecule. *Nature* **395**, 873–876 (1998).
[9] Blume-Kohout, R., da Silva, M. P., Nielsen, E., Proctor, T., Rudinger, K., Sarovar, M. & Young, K. A Taxonomy of Small Markovian Errors. *PRX Quantum* **3**, 020335 (2022).
[10] Bosco, S., Zou, J. & Loss, D. High-fidelity spin qubit shuttling via large spin-orbit interactions. *PRX Quantum* **5**, 020353 (2024).
